# Adolescent support club attendance and self-efficacy associated with HIV treatment outcomes in Tanzania

**DOI:** 10.1371/journal.pgph.0000065

**Published:** 2022-10-03

**Authors:** Gretchen Antelman, Ola Jahanpour, Thomas Machalo, Cosette Audi, Roland van de Ven, Mastidia Rutaihwa, Elise Lankiewicz, Sarah Baird

**Affiliations:** 1 Elizabeth Glaser Pediatric AID Foundation, Dar es Salaam, Tanzania; 2 Elizabeth Glaser Pediatric AID Foundation, Washington, DC, United States of America; 3 National AIDS Control Program, Ministry of Health, Dodoma, Tanzania; 4 Milken Institute School of Public Health, George Washington University, Washington, DC, United States of America; University of the Witwatersrand, SOUTH AFRICA

## Abstract

HIV treatment outcomes may be improved by ameliorating psychosocial challenges adolescents living with HIV (ALHIV) face. This analysis describes participation in existing facility-based adolescent clubs and the associations between club attendance, adolescent well-being and HIV treatment outcomes. Data were collected through interviews with a sub-sample of adolescents age 10–19 years and medical record abstraction of all adolescents attending HIV services at seven clinics in Tanzania. Independent variables included adolescent club attendance, self-esteem, self-efficacy, mental distress, social capital and other health utilization or HIV experience characteristics. Study outcomes included visit adherence, viral suppression (<1000 cp/ml), and retention. Of 645 adolescents attending HIV services, 75% attended clubs at least once with a median of eight club sessions attended over a two-year period. Mental distress was prevalent, with 67% of the adolescents scoring above a recognized cut-off of ≥5. Adolescents who attended 10 or more clubs, compared to those not attending any clubs over a two-year period were at an almost three-fold increased odds of having good visit adherence (odds ratio [OR] 2.72, 95% confidence interval [CI]: 1.25, 5.94). Club attendance was also associated with sustained retention in the following year: adolescents who attended some clubs (1–9) had three-times the odds of being retained (OR 3.01; 95%CI: 1.86, 4.87) while those who attended 10+ had over seven-times the odds (OR 7.29; 95%CI: 4.34, 12.22). Among the 154 adolescents who were interviewed, being in the top self-efficacy tertile was positively associated with viral suppression (OR 3.04, 95%CI: 1.08, 8.60) and retention (OR 4.44, 95%CI: 1.19, 17.40). Attending the HIV clinic with a guardian/treatment supporter (OR 3.29, 95%CI: 1.17, 9.22) was also associated with viral suppression. Self-efficacy, social capital and treatment support are associated with better health outcomes among adolescents. However, many ALHIV either never attended or did not regularly attend clubs.

## Introduction

More than 80% of the world’s 1.74 million adolescents living with HIV (ALHIV) reside in sub-Saharan Africa [1], representing 10% of all new HIV infections globally [2]. Viral suppression among adolescents is lower than adults living with HIV [3]. While many factors contribute to high viral load, poor adherence to antiretroviral therapy (ART) is considered a primary cause [4, 5]. Recent estimates of adherence among adolescents and young adults were approximately 50–60% in North America and Europe and 70% in Africa and Asia [6], compared to global rates of approximately 88% in adults [7].

During adolescence, individuals face a variety of challenges, from physical changes to evolving roles in their families, communities, and relationships. Adolescence is also associated with the onset of psychiatric disorders for some, and symptoms of depression/anxiety for more [8, 9], impacting an estimated 10–20% of adolescents in low and middle-income countries [10] and over 20% among ALHIV [8]. Emotional, behavioral, and psychiatric issues are more prevalent in many HIV-positive adolescent populations as compared to the general population [11], and these issues are often associated with lower levels of ART adherence [12–14].

Conversely, aspects of overall psychosocial well-being including self-esteem, self-efficacy and social capital have been positively associated with better health outcomes among ALHIV. In general, low self-esteem is considered to be a non-specific risk factor for mental health, associated with a variety of both mental and social problems [15]. Self-efficacy refers to one’s belief in their ability to behave intentionally in order to achieve particular results [16]. Some studies have associated self-efficacy with good ART adherence and viral suppression [17–20] and improved immunological measures [21], and self-efficacy is hypothesized to be a mediator between interventions and changes in health behavior across a broad spectrum of health issues [22]. Social capital describes "the networks of relationships among people who live and work in a particular society" [23]. In the context of ALHIV in sub-Saharan Africa, aspects of social capital that have been shown to be protective or to build resilience include social support from family, community and peers. Negative influences on social capital include stigma, and a disordered family/community environment due to death or other dysfunction [11, 24–26]. Access to financial or logistical support through social networks is also an important aspect of social capital associated with improved mental [11, 25, 27] and clinical outcomes for ALHIV [28].

Addressing mental and social well-being has become an important aspect of many adolescent HIV-related interventions. Diverse approaches [29] to psychosocial interventions among ALHIV have included enhancing education and counseling in HIV clinics [30]; engaging peers as individuals or establishing support clubs [31, 32]; and community and family-based [33] or clinic-based structured mental health interventions [34]. HIV peer support groups have been identified as providing numerous benefits to adolescents including social acceptance, reciprocal support, and personal growth and empowerment [35]. In Namibia, Munyayi and van Wyk [31] found no association between adolescent club attendance and ART adherence or viral suppression; but in a randomized trial in Zambia, adolescents assigned to a youth peer mentor intervention had significantly increased viral suppression compared to adolescents attending standard clinics [36]. While systematic reviews have generally found positive effects of support groups among ALHIV, they have also acknowledged the limited generalizability of these results due to differences in intervention and study designs [37].

This study focused on adolescents in the Arusha and Kilimanjaro regions of Tanzania. With an estimated 99,000 ALHIV, Tanzania ranks among the top five countries for new adolescent infections [1]. Previous analyses of ALHIV in the Kilimanjaro region found both significant prevalence of adverse mental health conditions and a positive association of mental health difficulties with incomplete adherence and experience of HIV-related stigma [14]. This study aims to describe the uptake and participation in facility-based ALHIV clubs and determine the association between club attendance and HIV outcomes: clinical visit adherence, viral suppression and retention. Secondary aims were to identify characteristics of well-being, social/treatment support and HIV knowledge associated with club attendance, and to explore how those characteristics relate to club attendance and HIV outcomes.

## Methods

### Ethics statement

This protocol was reviewed and approved by George Washington University’s Institutional Review Board (#071720) and the National Research Ethics Committee of the National Institute for Medical Research in Tanzania. Among those recruited for an interview, caregivers of minors and adolescents who were either emancipated or age 18 years or older provided written informed consent. Adolescent minors (age 10–17) provided verbal assent. For adolescents whose medical record information was included in the analysis but who were not recruited for interview, a waiver of informed consent was granted under a separate protocol approved by the National Research Ethics Committee of the National Institute for Medical Research in Tanzania and the US-based IRB, Advarra. All data were fully anonymized using participant and site ID numbers unlinked to any personal identifiers.

### Design, data sources and outcomes

We conducted a retrospective analysis of ALHIV attending seven health facilities with well-established adolescent clubs as part of their HIV clinical services. Data sources included (1) abstracted data on HIV/ART clinical visits from November 2015 to October 2019: (2) abstracted data on club attendance records from November 2016-October 2018; and (3) data collected from structured interviews with a subset of attending adolescents in January 2018. We interviewed the sub-sample of adolescents in order to describe and explore associations with other measures of adolescent well-being, including self-esteem, self-efficacy, social capitol, and mental distress (symptoms of depression/anxiety). We defined the visit adherence outcome from November 2016-October 2018, using data from the year prior as a baseline measure. Viral suppression was defined using laboratory results from the start of viral load testing services in 2017 up to October 2018. Finally, we defined a longer-term retention outcome which was measured up to October 2019. All adolescents aged 10–19 years old (as of October 31, 2017) who were retained in clinical HIV services during the first year of club attendance observation (November 2016-October 2017) were included in the study. A subset of adolescents who knew their HIV status were probability-selected from sex and age-group blocks (10–14 and 15–19 years) and recruited into the study for a structured interview.

### Adolescent club description and attendance

This study evaluated adolescent clubs which were already well-established at all study clinics prior to 2015 and independently run by clinic staff and management. Thus, the intervention is a real-world representation of a relatively mature, integrated, and sustainable service delivery model. At all facilities, clubs meet monthly on Saturdays when the HIV clinic is otherwise closed to general patients, and comprehensive HIV care is provided at the club meetings by clinical, laboratory and pharmacy staff. In addition, refreshments are offered and some form of “edutainment” takes place (games, songs, dramas). During the study, the recommended visit frequency was monthly so most patients attending HIV care were expected to have about 12 visits annually. However, in practice, different factors may have contributed to non-regular visit and club attendance including multi-month prescriptions; attending during regular clinic hours (not Saturdays) with treatment supporters; having treatment supporters collect ARVs for them (common for adolescents at boarding schools); or just missing visits in their schedule. Therefore, we categorized club attendance into three groups, roughly defined by the tertile distribution of observed club attendance over the 2-year period: non-attenders (0 clubs), non-regular attenders (1–9 clubs) and regular attenders (10 or more). We chose not to treat club attendance as a continuous independent variable because we hypothesized that more regular attendance to clubs was a threshold above which an association with health outcomes could be observed. However, our analysis of factors associated with club attendance (as a dependent variable) retained the measure original continuous form.

In early 2016, three facilities adopted a slightly different club model whereby ALHIV age 18 or older were purposely selected by the facility staff to become trained peer facilitators responsible for leading adolescent club meetings, presenting on topics related to positive living with HIV such as adherence, treatment literacy, managing stigma, disclosure, sexual/reproductive health, and transition to adult care. At the other four facilities, club meetings, led by clinic staff, were oriented toward a wider age group that included children under age 10. Thus, the health talks were focused on a narrower set of HIV-related topics, with less emphasis on information about sexual/reproductive health or adolescent transition to adult care. Our analyses did not show that club attendance or outcomes differed by club model, therefore, we adjusted multivariable models by facility (cluster).

### Data collection

Medical records were abstracted to obtain information about all clinical visits as well as those on Saturdays indicating club attendance, HIV viral load test results, dates of birth and ART initiation. In order to maximize completeness and accuracy, club attendance was also abstracted from club attendance lists. Any adolescent noted on the club attendance list or having a clinic visit on a Saturday in the visit database was counted as having attended a club. The maximum number of club meetings attended and/or Saturday visits was taken as the annual total number of clubs attended for this analysis.

The subset of adolescents recruited into the study were interviewed by trained research assistants using a structured questionnaire that included questions about household characteristics, recent ART adherence (self-reported missed dose); HIV/ART knowledge; characteristics of visit attendance (transport, treatment supporter); HIV disclosure; and psychosocial well-being and support measures described below (S1 and S2 Texts). The 30–40 minute interview was conducted in a private setting, using the local Swahili language. Data were double-entered (Epi-Info; https://www.cdc.gov/epiinfo/index.html) and discrepancies verified and corrected. Participants in the interview were identified only by a unique study identification number on the interview document and in the database.

### Measures

#### Outcome variables: Visit adherence, viral suppression and retention

Visit adherence was classified as a binary variable, dichotomized as “good” versus “poor.” At each clinical visit, the clinician documents how many days of ARVs are provided, and the next (typical monthly) appointment is set about 2–3 days prior to the date the patient would run out of ARV drugs. If the patient misses the appointment altogether or returns long after they have run out of drug, they are classified as having poor adherence to their visit schedule. Adolescents were defined as having good visit adherence (over a defined time period) if they kept close enough to their appointments that they never had more than a 5-day gap between running out of their ARV drugs and their next visit.

Viral suppression was defined as having a viral load below 1000 copies/milliliter (cp/ml), based on the latest viral load result up to October 2018. Participants missing a viral load measure during this time period (n = 109; 17%) were excluded from the suppression analysis. Sensitivity analyses imputing missing values to suppressed versus not suppressed, as well as cross-tabluation of viral load missingness with visit adherence and retention outcomes are shown in supporting information (S1 Table).

Adolescents who remained on ART within 90 days of October 31, 2019 were classified as retained, including verified transfers from one clinic to another. Those reported to have died or missed ART refill visits ≥90 days before October 2019 were classified as non-retained, including unverified transfers.

#### Independent variables

*Self-esteem*. The Rosenberg self-esteem scale (RSES), developed to measure the self-esteem of high schoolers (Rosenberg, 1965), was used to measure one aspect of adolescent well-being. Each item of the 10-item scale is assigned one of four values from “strongly agree” to “strongly disagree.” The scale sums to values between 0 and 30, with a higher value indicating higher self-esteem. Low self-esteem has been associated with depressive symptoms and social anxiety in adolescents [38].

*Self-efficacy*. The HIV Treatment Adherence Self-Efficacy Scale (HIV-TASES) was used to assess adherence integration and perseverance [39]. Each of twelve questions were scored from 0 to 10, with zero indicating no confidence in the ability to perform the adherence related activity and 10 equating to complete confidence in their ability. This results in a scale from 0 to 120, with higher scores indicating higher self-efficacy.

*Mental health*. The 12-item General Health Questionnaire (GHQ-12) includes items assessing the respondent’s ability to concentrate/sleep, manage stress, make decisions, maintain daily activities, problem solve, and to what degree they felt useful, happy or confident over the past four weeks. Items are scored on a 4-point scale; however bimodal scoring, commonly used in clinical settings for rapid screening outcomes, was used. Thus, each item is scored as “0” for responses of “less than” or “no more than usual” and “1” for responses “rather more” and “much more than usual.” The GHQ-12 has been validated internationally among primary care patients [40, 41] and young adults [42]. The GHQ-12 was one of two recommended tools for common mental disorder screening by non-medical interviewers [43], and a recent study in Nigeria noted that the GHQ had fewer false positives compared to the Patient Health Questionnaire [44]. Cut-offs vary across studies but are generally agreed to be ≥5 for the 0–12 bimodal scoring, indicating moderate/severe distress [42]. These measures of well-being were analyzed in regression models as categorical variables, divided according to reported cut-offs for self-esteem and the GHQ-12, and into tertiles for self-efficacy.

*HIV disclosure*, *knowledge and social capital*. Interviewed adolescents were asked at what age they were when they were told they had HIV, who disclosed to them, and whether they had suspected they had HIV before they were disclosed to. We also asked whether they had disclosed to anyone outside of the HIV clinic, and whether their guardian or treatment supporter usually attended the clinic with them. We assessed HIV knowledge with questions about ARV effects on transmission risk, the meaning of a low CD4 count (this was prior to widely available viral load testing), and general modes of transmission. Three social capital variables were defined according to perceived access to any of three types of support: emotional support; connection to their community; and financial/logistical support.

### Statistical analyses

Descriptive statistics were produced for all variables used in the analysis and differences were described between those who participated in interviews and those who did not. After examining bivariate relationships between our predictor and outcome variables using non-parametric tests (Chi-square, Wilcoxon Rank Sum), we built multivariable models, adjusting for facility, age and sex. Club attendance was defined as none, 1–9 and ≥10 clubs attended over the two-year period (roughly the median cut-off among club attenders). Logistic regression models were built separately for the whole study group and for the subset of those interviewed, where additional adolescent characteristics and measures of well-being were examined. Ordinary least squares regression analysis was used to identify factors associated with the number of club sessions attended. Stata (version 16.1, https://www.stata.com/) was used for all statistical analyses.

## Results

Table 1 compares characteristics and outcomes for all adolescents (n = 645) stratified by whether they participated in the study interview or not. The median age of all adolescents was 14 years (inter-quartile range [IQR] 12,17), median ART duration was 6 years (IQR 3,9), and just over half (53%) were male. The subset of adolescents interviewed for the study were more likely to have attended adolescent club meetings over the two-year observation period compared to those not interviewed (median 14 versus 6; p < .0001) and had more clinic visits (median 21 versus 18; p < .0001). There were no significant differences between the two adolescent groups in viral suppression. Good visit attendance over the two-year period (16% versus 10%; p = .022) and retention (85% versus 66%; p < .0001) were significantly higher among those interviewed compared to those not interviewed.

**Table 1:** Characteristics and study outcomes for all adolescents attending study sites (n = 645), stratified by whether they participated in the interview (n = 154) or not (n = 491).

	All adolescents (n = 645)	Interviewed adolescents (n = 154)	Not interviewed adolescents (n = 491)	p-value*
	N (%) or Median (inter-quartile range [IQR])			
Male	339 (53%)	77 (50%)	262 (54%)	0.47
Female	306 (47%)	77 (50%)	229 (46%)	
Age in years	14 (IQR: 12, 17)	15 (IQR: 12, 17)	14 (IQR: 12, 16)	0.14
Duration on ART in years	6 (IQR: 3, 9)	7 (IQR: 4, 9)	6 (IQR: 3, 9)	0.23
**Two-year observation period**				
**Number club meetings attended** (continuous)	8 (IQR: 1, 17)	14 (IQR: 5, 20)	6 (IQR: 0, 15)	<0.0001
**Number club meetings attended** (categorical)				<0.0001
None	150 (23%)	18 (12%)	132 (27%)	
1–9 meetings	204 (32%)	42 (33%)	162 (27%)	
10–19 meetings	197 (30%)	53 (34%)	144 (29%)	
20+ meetings	94 (15%)	41 (27%)	53 (11%)	
N**umber of clinical visits** (continuous)	19 (IQR: 12, 22)	21 (IQR: 16, 23)	18 (IQR: 10, 22)	<0.0001
N**umber of clinical visits** (categorical)				<0.0001
<10 visits	121 (19%)	11 (7%)	110 (22%)	
10–19 visits	234 (36%)	52 (34%)	182 (37%)	
20+ visits	290 (45%)	91 (59%)	199 (41%)	
**Outcomes**				
**Good visit adherence** **				
Year 1: November 2016-October 2017	164 (25%)	53 (34%)	111 (23%)	0.003
Year 2: November 2017-October 2018	203 (31%)	55 (36%)	148 (30%)	0.19
Two-year period	72 (11%)	25 (16%)	47 (10%)	0.022
**Viral suppression**				0.57
Suppressed (<1000 cp/ml)	304 (55%)	81 (55%)	223 (57%)	
Not suppressed (1000+)	232 (45%)	67 (45%)	165 (43%)	
No viral load measure (excluded)	109 (--)	6 (--)	103 (--)	
**Retention**				< .0001
Retained at site, or verified transfer	453 (70%)	131 (85%)	322 (66%)	
Not retained (lost to follow-up or died)	192 (30%)	23 (15%)	169 (34%)	

* P-values determined using non-parametric tests comparing interviewed versus not-interviewed adolescents (Chi-square for categorical and Wilcoxon Rank Sum Test for continuous variables)

**Not missed any ART refill visit by 5+ days

HIV care-seeking, disclosure, knowledge and measures of well-being collected from the subset of interviewed adolescents are shown in Table 2. One-third (31%) are accompanied to the clinic by their guardian/treatment supporter, and more than half (58%) were told they had HIV when they were between the ages of 10–14 years, with 15% being told at 15 years or older. Just over one-third (39%) had disclosed their HIV positive status to a friend/partner (excluding those in the HIV clinic setting), and 17% reported suspecting they had HIV before they were disclosed to themselves. Most adolescents were able to correctly answer different HIV knowledge questions (range across measures 57%-86%). Reported levels of social capital were high, ranging from 84% for financial support to 100% for emotional support. Self-esteem and self-efficacy median scores were 22 (IQR 20,24) and 105 (IQR 90,111) respectively, with a very low proportion of adolescents falling below the established cutoff of 15 classified as low self-esteem (4%). Mental distress (symptoms of depression and anxiety) was prevalent, with 67% of the adolescents scoring above a recognized cut-off of ≥5.

**Table 2:** Characteristics of HIV care-seeking, disclosure, knowledge and measures of well-being assessed among the subset of adolescents enrolled and interviewed (n = 154).

	Interviewed adolescents (n = 154)
Guardian/Treatment supporter attends clinic with adolescent	47 (31%)
Suspected having HIV before being disclosed to	26 (17%)
**Age when disclosed to**	
<10 years	39 (27%)
10–14	84 (58%)
15+	22 (15%)
Disclosed HIV status to another (exclude medical provider)	60 (39%)
**How concerned someone not disclosed to will discover your status**	
Very concerned	91 (61%)
A little concerned	23 (16%)
Not concerned	34 (23%)
**Answered correctly to knowledge questions:**	
Taking ARVs can reduce transmission risk	115 (75%)
Low CD4 is bad for health	133 (86%)
Pregnant woman can transmit HIV	88 (57%)
Breastfeeding can transmit HIV	123 (80%)
HIV transmission possible from someone looks healthy	111 (72%)
**Social Capital**	
Emotional support/guidance^1^	154 (100%)
Social belonging^2^	144 (94%)
Financial/logistical support^3^	129 (84%)
	**Median (IQR)**
Rosenberg **Self-esteem** scale (10 items; range 0–30)	22 (IQR: 20, 24)
Percent having low self-esteem, cutoff score = 15	6 (4%)
HIV Adherence and **Self-efficacy** scale (12-items; range 0–120)	104 (IQR: 90, 111)
General Health Questionnaire score (**GHQ 12**, bimodal scoring; range 0–12)	6 (IQR: 4, 6)
Percent above cut-off ≥5	103 (67%)

Social capital questions (7) were grouped into the following variables, defined as the proportion of items the respondent answered as “agree” and “strongly agree” to at least one in the group

^1^ *Emotional*: Is there someone to talk to (1) if confused/disappointed, (2) if upset, (3) about an important problem, (4) for guidance

^2^ *Social*: Does respondent (4) perceive community trusts them, (5) feels trust in community, (6) perceive neighbors comfortable sharing problem with them)

^3^ *Financial*: (7) Can respondent access emergency financial/other help if needed

### HIV outcomes–visit adherence, viral suppression, retention

The odds associated with attending 10 or more clubs over the two-year period were estimated using maximum-likelihood dichotomous logistic regression on three HIV outcomes, adjusted for facility, age, sex and duration on ART (Table 3) for the all-adolescent sample and the sub-sample of interviewed adolescents. Adolescents who attended 10 or more clubs over two years, compared to those not attending any clubs, were at an almost three-fold increased odds of having good visit adherence (odds ratio [OR] 2.72, 95% confidence interval [CI]: 1.25, 5.94; all-adolescent sample). Any club attendance over a two-year period was also strongly associated with retention in the following year, with adolescents attending some clubs (1–9) having three-times the odds of being retained (OR 3.01; 95%CI: 1.86, 4.87) and adolescents attending more frequently (10+) having over seven-times the odds (OR 7.29; 95%CI: 4.34, 12.22). No association between club attendance and viral suppression was observed, but the sensitivity analysis suggested an association with club attendance (10+ clubs) and viral suppression, if those who were missing viral load measures were indeed less likely to be suppressed, consistent with the observation that viral load missingness was 2–3 times less likely among those with good visit adherence and retention (see supplementary material).

**Table 3:** Facility-adjusted* logistic regression modelling of associations between adolescent club attendance and study outcomes among all adolescents attending study sites and the subset of adolescents enrolled and interviewed.

	Good visit adherence (not missed any ART refill visit by 5+ days)		Viral suppression** (viral load <1000 cp/ml)		Retention	
	Odds ratio [OR] (95% CI)	p-value	OR (95% CI)	p-value	OR (95% CI)	p-value
**All adolescent model (n = 645)**						
**Club attendance**						
None	1.00		1.00		1.00	
1–9 club meetings	0.61 (0.24, 1.58)	0.31	0.70 (0.41, 1.20)	0.19	3.01 (1.86, 4.87)	< .0001
≥10 club meetings	2.72 (1.25, 5.94)	0.012	1.12 (0.66, 1.88)	0.68	7.29 (4.34, 12.22)	< .0001
Male sex (ref: F)	1.05 (0.63, 1.76)	0.84	0.97 (0.68, 1.38)	0.87	0.99 (0.68, 1.45)	0.96
Age in years	1.01 (0.92, 1.12)	0.77	0.93 (0.87, 1.00)	0.045	0.89 (0.83, 0.96)	0.001
Years on ART	--	--	0.92 (0.87, 0.97)	0.002	--	--
**Subset interviewed adolescents (n = 154)**						
**Club attendance** (Nov2016-Oct2018)						
None	1.00		1.00		1.00	
1–9 club meetings	0.24 (0.03, 1.75)	0.16	0.54 (0.11, 2.73)	0.46	2.41 (0.51, 11.43)	0.27
≥10 club meetings	1.26 (0.27, 5.98)	0.77	2.42 (0.49, 11.81)	0.28	3.98 (0.80, 19.78)	0.09
**Self-efficacy tertile**	--	--				
Low (ref)			1.00		1.00	
Medium			2.12 (0.78, 5.73)	0.14	2.31 (0.68, 7.91)	0.18
High			3.04 (1.08, 8.60)	0.036	4.44 (1.19, 17.40)	0.027
Male sex (ref: F)	0.87 (0.35, 2.18)	0.77	0.67 (0.30, 1.54)	0.35	1.18 (0.43, 3.27)	0.75
Age in years	0.98 (0.82, 1.18)	0.86	1.01 (0.86, 1.19)	0.89	0.92 (0.76, 1.13)	0.43
Years on ART			0.89 (0.78, 1.01)	0.069		
**Poor adherence indicator** (missed ARV dose in past 7 days)	--	--	0.23 (0.09, 0.57)	0.001	--	--
**Guardian/treatment supporter attends clinic with adolescent** (ref: adolescent attends on own)	--	--	3.29 (1.17, 9.22)	0.024		

* All facilities included in model as dummy variable.

** Due to missing viral load results, analytic samples for viral suppression included n = 536 and n = 148 adolescents, respectively

Among the subset of interviewed adolescents, adolescents scoring in the high self-efficacy (SEFF) tertile had over four times higher odds of retention (OR 4.44, 95%CI: 1.19, 17.40) and over three times higher odds of viral suppression (OR 3.04, 95%CI: 1.07, 8.60), though there was no observed association between self-efficacy and visit adherence. Poor adherence had a strong negative association with viral suppression (OR 0.23, 95%CI 0.09, 0.57). Attending the HIV clinic with a guardian/treatment supporter (OR 3.29, 95%CI: 1.17, 9.22) was associated with viral suppression but was not associated with the other study outcomes (Table 3). Low self-esteem was not associated with any study outcome.

### Club attendance

Nearly one-quarter of adolescents did not attend any club visits during the observation years (23%), and only 15% attended almost monthly (20+ club visits) over the two-year period (Table 1). We used ordinary least squares regression to identify factors associated with club attendance (Table 4). Visit adherence patterns established in the year prior to the study period (November 2015-October 2016). Those with good visit adherence attended 2.64 more clubs compared to those with poor visit adherence (regression coefficient (B_1_) 2.64, 95%CI: 1.30, 3.97). Among the sub-sample of interviewed adolescents, having a social capital score above the median was associated with attending 3.24 more clubs (B_1_ 3.24, 95%CI 0.64, 5.85), thus.

**Table 4:** Factors associated with annual number of club meetings attended: Facility*, sex and age adjusted linear regression.

	All adolescents n = 645		Interviewed adolescents n = 154	
	Beta (95% CI)	p-value	Beta (95% CI)	p-value
**Good visit adherence** (in year preceding the study observation period for club attendance)**	2.64 (1.30, 3.97)	<0.0001	3.06 (0.66, 5.45)	0.013
Male sex (ref: F)	0.31 (-0.85, 1.46)	0.60	2.56 (0.29, 4.83)	0.027
Age in years	-0.31 (-0.52, -0.09)	0.005	-0.56 (-1.00, -0.10)	0.017
**Social Capital**	--	--		
Below median			1.00	
Above median			3.24 (0.64, 5.85)	.015

* All facilities included in model as dummy variable

**Not missed any ART refill visit by 5+ days

## Discussion

This study supports the need for comprehensive psychosocial support interventions for ALHIV with mental health assessment and service scaffolding to diagnose and address adolescents’ needs. Our finding that regular adolescent club attendance is associated with visit adherence and retention is consistent with other studies on the effects of peer group interventions for adolescents [31, 34, 36, 45]. A recent review of such interventions described several potential mechanisms for how adolescent clubs support HIV-related outcomes [35]. The clubs are designed to offer routine and integrated HIV clinical and psychosocial support services in a flexible and continuous manner, adapting to adolescent needs for information and support as the group members mature or face particular challenges. Peer and health provider facilitators trained in adolescent-friendly services promote non-judgmental interactions with club attendees. Being in a separate space with their peers to share common life experiences related to disclosure, managing one’s health, and relationships with family and friends contributes to an adolescent’s social capital and sense of self-efficacy [35]. While we would have expected to find an indirect effect of both better visit adherence and retention on viral suppression, through the mechanisms discussed above, we did not detect significant associations between adolescent club attendance and viral suppression. This may be due to the small sample size among those interviewed, and a high rate of missingness among the all-adolescent group (refer to supplementary material for further details).

Few studies of adolescent club interventions have reported on the uptake or regularity of club attendance, or described factors associated with club attendance. We found that about one-quarter of adolescents do not attend the clubs at all over a two-year period, one-third attend at sub-optimal frequency and just under half attend frequently. Thus, even when effective group/peer-based interventions are available, the majority of adolescents will not benefit due to lack of participation. While we identified some factors associated with club attendance, such as having higher social capital or good visit adherence, we cannot conclude that these characteristics are a cause or effect of club attendance since most of the adolescents in our study were long-standing ART patients and had established patterns of club attendance prior to this study. To address this gap in the literature, formative smaller-scale observational and qualitative research should aim to identify characteristics of non-club attendees to inform interventions to better engage those adolescents. Larger randomized multi-center trials are needed to answer questions about uptake of psychosocial support interventions among newly enrolled versus well-established-on-ART adolescents, as well as the potential psychosocial mechanisms of club effectiveness. Furthermore, multi-month prescription to stable ART patients has recently become the standard of care. Clinic/refill visits spaced every 4–6 months could pose a barrier to facility-based adolescent clubs built around the model of integrated ART service delivery. As ART services evolve, providers should consider how ART adherence and psychosocial/peer support could take place in alternative venues, and how to further empower peer facilitators to lead community-based support groups. Further research is needed to determine optimal levels of club attendance in terms of frequency and duration. For example, are clubs most beneficial on a monthly schedule, or would intensive periods of more frequent attendance from which adolescents “graduate” after a number of sessions also support positive health outcomes?

Among the measures of well-being explored in our study, only self-efficacy was found to be associated with viral suppression and retention. We found only one study from sub-Saharan Africa on how adolescent self-management related to ART adherence and viral suppression [17], a concept similar to self-efficacy that included HIV treatment knowledge and confidence to manage illness and health care services, motivation and self-advocacy to obtain necessary social support from others, community participation, and coping/self-regulation with regard to managing stigma and disclosure decisions. Self-efficacy has been linked to enhancing resilience against negative influences of mental distress or stigma. A study among adults in the United States (U.S.) and Puerto Rico found self-efficacy was strongly related to ART adherence behaviors, and partly mediated the negative influences of lack of social capital or mental distress on ART adherence [46]. And in a smaller study of U.S. adults, self-efficacy was described as a mediating factor in the relationship between reduction in internalized stigma and improved viral suppression, ART and visit adherence [47]. In our analysis, adjusting for poor adherence–a robust independent factor negatively associated with viral suppression–reduced but did not eliminate the association between self-efficacy and viral suppression, supporting prior findings that self-efficacy is a broader construct of well-being or resilience that is important to HIV treatment outcomes.

Adolescents with guardians or treatment supporters who attend the clinic with them were more likely to be virally suppressed, while social capital, a construct that may overlap with treatment support, was not. Having robust treatment support is consistent with a large population-based study among adolescents in South Africa finding a protective effect of parental/caregiver engagement on adherence [48]. Engagement of treatment supporters in an adolescent’s clinical visit is likely linked to better knowledge/understanding around managing ART with regard to dosage, timing, and side-effects, as well as facilitating supportive routines in the home for maintaining adherence and greater openness of communication between the adolescent and guardian around living with HIV generally.

While we did not find that mental distress, as measured by the GHQ-12, was associated with HIV outcomes or adolescent club participation, we did observe high levels of psychological distress, with two-thirds of the adolescents interviewed scoring above a recognized cut-off of ≥5 indicating moderate or severe distress. This finding of high levels of mental distress is consistent with other studies among ALHIV finding prevalence of 13% in Tanzania [14] and over 30% among youth in Ethiopia [49]. There is a need for HIV services to incorporate mental health screening and interventions into adolescent HIV services [10]. While adolescent club participation is linked to better HIV outcomes, comprehensive mental health interventions will require significantly greater investment, standardization, and professional engagement.

### Limitations

This study had several limitations. First, this was a descriptive analysis and thus causality was not established. Second, club attendance varied substantially by facility suggesting that adolescent clubs are likely implemented differently across clinics, and that there were important clinic-related factors that were not measured in this study, such as clinic leadership and staff commitment to adolescent services. We did collect data on distance/cost to clinic and household socio-economic security, finding these unrelated to club attendance. However, other patient or family-related factors that could have explained some of this variation, such as educational or employment commitments, were not measured. Third, this study’s use of routine program data mean that our visit adherence outcome, retention and club attendance measures may be imprecise. Visit adherence was a variable derived from documentation on the number of ART tablets dispensed which may not always be complete or accurate. If documentation of ART refills from other clinics or longer multi-month prescriptions were missing in the database, we will have over-estimated non-adherence and non-retention and reduced the likelihood of detecting associations between factors of interest and adherence/retention. Participation in clubs may have been under-counted due to poor record keeping of club attendance, thereby under-estimating the association between club attendance and study outcomes. A relatively high degree of missing viral load measures also reduced our power in the viral suppression analysis. A fourth limitation is that the sub-sample of adolescents enrolled for interview differed from the larger group on many characteristics; plus our site selection was not random nor was the study large enough to include more regions and clinic settings. And finally, the dichotomization of club attendance may not be transferable to other settings and populations. Our rationale for the cut-point of 10 clubs was based on the recommended clinic visit schedule in Tanzania and the distribution of our data. These factors limit the generalizability of our findings to the broader ALHIV population.

## Conclusion

This study adds to the growing body of evidence of the role of both adolescent clubs and broader overall psychosocial well-being in improving outcomes for ALHIV, highlighting the importance of adolescent clubs and self-efficacy on visit adherence, viral suppression and retention in HIV care. One strength of this study is that it evaluated adolescent clubs as they are offered in real-world HIV services rather than as a controlled research intervention. However, this means that the magnitude of association between club attendance and HIV outcomes will vary depending on how well any clinic’s club is addressing and adapting to adolescent needs in their patient population. This points to the need for guidelines and toolkits that support implementation of group-based, peer-facilitated clubs and integration of mental health components into HIV service delivery [50], similar to those already existing for disclosure [51] and transition [52]. Such tools would facilitate standardized in-country training, guidelines, policies and documentation and monitoring; and support programs to better define adolescent-friendly services and implement interventions for youth living with HIV with fidelity.

## Supporting information

S1 Text Adolescent structured questionnaire in English. (DOCX)

S2 Text Adolescent structured questionnaire in Swahili. (DOCX)

S1 Table Explores the association between club attendance and viral load under different imputation assumptions around those with missing viral loads (all suppressed, none suppressed). (DOCX)
