# Supplementary material for: Adolescent support club attendance and self-efficacy associated with HIV treatment outcomes in Tanzania
Source: PLOS Glob Public Health. 2022 Oct 3;2(10):e0000065. doi: 10.1371/journal.pgph.0000065 (PMC10021176; doi:10.1371/journal.pgph.0000065)
Supplement: S1 Text — (DOCX) [file pgph.0000065.s001.docx]

**Adolescent Structured Questionnaire**

|  |  |  | **1 male**  **2 female** |
| --- | --- | --- | --- |
| **DATE** | **RA CODE** | **PTID** | **SEX** |
|  |  |  |  |

| Interview start time: |  |  |  | : |  |  |  |
| --- | --- | --- | --- | --- | --- | --- | --- |
|  | | hour | | minute | | | |

| **A. HOUSEHOLD BACKGROUND AND DEMOGRAPHICS** | | | | | | | | | |
| --- | --- | --- | --- | --- | --- | --- | --- | --- | --- |
| **no** | **questions and filters** | | **coding categories** | | | | | **skip** | |
| **A01** | What region do you currently live in? | | arusha  kilimanjaro | | | **1**  **2** | |  | |
| **A02** | How long have you lived in your current residence? | | \| years \|  \|  \| \| --- \| --- \| --- \| \| if < 1 year \| **00** \| \| | | | | |  | |
| **A03** | Including yourself, how many people live in your household right now? | | \| people \|  \|  \| \| --- \| --- \| --- \| | | | | |  | |
| **A04** | Who is your primary guardian? | | Biological parent  Grandparent  Other elder relative (sister, aunt, uncle, etc.)  Non-relative (neighbor, friend, community person)  Other, specify ____________________ | | | **1**  **2**  **3**  **4**  **5**  **6** | |  | |
| **A05** | What is the sex of your primary caregiver? | | male  female | | | **1**  **2** | |  | |
| **A06** | Do you or any member of your household own a…  read all items aloud and circle a response for each one | \|  \| **yes** \| **no** \| **dk** \| \| --- \| --- \| --- \| --- \| \| radio \| **1** \| **2** \| **8** \| \| television \| **1** \| **2** \| **8** \| \| mobile telephone \| **1** \| **2** \| **8** \| \| refrigerator \| **1** \| **2** \| **8** \| \| bicycle \| **1** \| **2** \| **8** \| \| motorcycle \| **1** \| **2** \| **8** \| \| animal drawn cart or donkey(s) \| **1** \| **2** \| **8** \| \| Car/Truck \| **1** \| **2** \| **8** \| | | | | | |  | |
| **A07** | What is the main source of energy for lighting in the household? | | ELECTRICITY  SOLAR  GAS  PARAFFIN LAMP  OTHER | | **1**  **2**  **3**  **4**  **7** | | |  | |
| **A08** | What is the main source of drinking water for members of your household? | | PIPED WATER  DUG WELL  WATER FROM SPRING  BOTTLED WATER  OTHER | | **1**  **2**  **3**  **4**  **9** | | |  | |
| **A09** | What kind of toilet facility do members of your household usually use? | | FLUSH OR POUR FLUSH TOILET  PIT LATRINE  OTHER | | **1**  **2**  **7** | | |  | |
| **A10** | What type of fuel does your household mainly use for cooking? | | ELECTRICITY  BOTTLED GAS  PARAFFIN/KEROSENE  CHARCOAL  FIREWOOD  OTHER | | **1**  **2**  **3**  **4**  **5**  **8** | | |  | |
| **A11** | What type of flooring does your home have? | | EARTH/SAND  CEMENT  TILES  OTHER | | **1**  **2**  **3**  **4** | | |  | |
| **A12** | What type of the walls or your house made of? | | | Cement  Brick (matofali ya kuchoma)  Mud brick (matofali mabichi)  Mud (makuti)  Other ______________________________ | | | **1**  **2**  **3**  **4**  **5** | |  |
| **A13** | What type of roof of your house made of? | | | Tin / tile  Grass  Other -__________________________ | | | **1**  **2**  **3** | |  |
| **A14** | Can you tell me how many cows your family own? | | | <5 (talk to Daniel Masai; Chrispine?)  5-9  10-19  20+ | | | **1**  **2**  **3**  **4** | |  |
| **A15** | Can you tell me how many sheeps/goats your family own? | | | <5 (talk to Daniel Masai; Chrispine?)  5-9  10-19  20+ | | | **1**  **2**  **3**  **4** | |  |
| **A16** | How often in the past six months did your household have trouble satisfying its food needs? Would you say never, seldom, sometimes, often or always? | | | never  seldom  sometimes  often  alway | | | **1**  **2**  **3**  **4**  **5** | |  |

| **A17** | How old are you in complete years?  enter year born or age in years, but not both. probe to nearest 5 years if respondent cannot report age or year of birth | \| age in years \|  \|  \| \| --- \| --- \| --- \| | |  |
| --- | --- | --- | --- | --- | --- | --- | --- |
| **A18** | What is the highest level of formal schooling you completed? | None  Some Primary  Completed Primary  Some Secondary  Completed Secondary  Higher (College, University, and above) | **1**  **2**  **3**  **4**  **5**  **6** |  |
| **A19** | Have you ever been married? | Yes, currently married  Yes, Divorced  Yes, Widowed  No | **1**  **2**  **3**  **4** | **>>A16** |
| **A20** | If yes, how old were you when you first got married? | Age in Years   \|  \|  \| \| --- \| --- \| |  | **>>A18** |

| **A21** | Have you ever been in a sexual relationship? | YES  NO | **1**  **2** |  |
| --- | --- | --- | --- | --- |
| **A22** | Have you ever had sex? | Yes  No | **1**  **2** |  |
| **A23** | If yes, how old were you for your first sexual experience? | Age in Years   \|  \|  \| \| --- \| --- \| |  |  |
| **A24** | What was the age difference in years between you and your first sexual partner? | <2 Y  2-4  5-9  10+ | **1**  **2**  **3**  **4** |  |
| **A25** | Have you ever had a non-consensual sexual encounter? That is, any sexual encounter with a person who pressured or forced you when you wanted to say no? | Yes  No | **1**  **2** |  |
| **A26** | Have you ever been pregnant? | Yes  No | **1**  **2** | **>>Next Section** |
| **A27** | If yes, how old were you for your first pregnancy? | Age in Years   \|  \|  \| \| --- \| --- \| |  |  |

| **B. HIV CARE AND KNOWLEDGE** | | | | |
| --- | --- | --- | --- | --- |
| **no** | **questions and filters** | **coding categories** | | **skip** |
| **B01** | How old were you when you first entered HIV care? | \| age in years \|  \|  \| \| --- \| --- \| --- \| | |  |
| **B02** | How long have you been attending your current HIV clinic (CTC)? | \| months \|  \|  \| \| --- \| --- \| --- \| \| years \|  \|  \| \| don’t know \| **88** \| \| | |  |
| **B03** | How many different clinics did you attend before this one? | \| Number \|  \|  \| \| --- \| --- \| --- \| | |  |
| **B04** | Have you started taking ARVs? | yes  no  don’t know | **1**  **2**  **8** |  |
| **B05** | When did you first take ARV medicines?  enter months, years ago | \| months ago \|  \|  \| \| --- \| --- \| --- \| \| years ago \|  \|  \| \| don't know \| **88** \| \| | |  |
| **B06** | How old were you when you first took ARV medicines? | \| age in years \|  \|  \| \| --- \| --- \| --- \| | |  |
| **B07** | Are you still taking ARV medicines? | yes  no  don’t know | **1**  **2**  **8** |  |
| **B08** | In the past 7 days, how many days did you miss taking one or more of your ARV medicine pills? | days one or more pills were not taken: _____  Don’t know | **8** |  |
| **B09** | Some people find that they may go without pills for 2 or more consecutive days. Have you ever missed taking ALL of your ARV medicines for 2 or more consecutive days? | yes  no  don’t know | **1**  **2**  **8** |  |
| **B10** | Do you have a treatment supporter, that is, someone in your household who helps you remember to take your medicine and attend the clinic? | yes  no  don’t know | **1**  **2**  **8** |  |
| **B11** | Does your guardian/treatment supporter attend the CTC for his/her own HIV care? | yes  no, not HIV positive  no, HIV positive, but not attending care  don’t know | **1**  **2**  **3**  **8** |  |
| **B12** | Does your usually guardian/treatment supporter attend the CTC with you or do you attend on your own? | On Own  With Guardian/Treatment Supporter  Other | **1**  **2**  **3** |  |
| **B13** | What type of transport do you usually use to come to the clinic? | Walk  Bicycle taxi  Boda boda (motorcycle)  Dala Dala (mini bus)  Taxi  Own/borrowed bicycle  Own car  Other (specify)__________ | 1  2  3  4  5  6  7  8 |  |
| **B14** | How much did you pay for transport to or from for your most recent appointment?  count cost for one-way only. if costs vary for going to ctc vs coming from ctc, choose highest cost | \| Tanzania shillings \|  \| \| --- \| --- \| \| don’t know \| **888 888** \| \| refuse to answer \| **999 999** \| | | |
| **B15** | Approximately how long did it take you to get from your home to the clinic for most recent appointment | \| HOURS:MIN \|  \|  \| **:** \|  \|  \| \| --- \| --- \| --- \| --- \| --- \| --- \| \|  \|  \| \| \| \| \| | | |
| **B16** | Over all your visits, how much time do you usually spend at clinic from the time you arrive to the time you leave? | less than 1 hour  1 - 3 hours  4 - 6 hours  more than 6 hours  don’t know  refuse to answer | **1**  **2**  **3**  **4**  **8**  **9** |  |
| **B17** | When a person with HIV begins taking ARV medicines, does his or her risk of giving HIV to a sexual partner increase, decrease, or remains the same? | increase  decrease  remains the same  don’t know | **1**  **2**  **8** |  |
| **B18** | Is having a low CD4 count, good or bad for your health? | good for health  bad for health  don’t know | **1**  **2**  **8** |  |
| **B19** | A pregnant woman can transmit the AIDS virus to her unborn child | yes  no  don’t know | **1**  **2**  **8** |  |
| **B20** | A woman can transmit AIDS virus to her child through her breast milk | yes  no  don’t know | **1**  **2**  **8** |  |
| **B21** | You can get AIDS virus if you have sex with someone who looks perfectly healthy | yes  no  don’t know | **1**  **2**  **8** |  |
| **B22** | AIDS has a cure | yes  no  don’t know | **1**  **2**  **8** |  |

| **C. DISCLOSURE** | | | | | |
| --- | --- | --- | --- | --- | --- |
| **no** | **questions and filters** | | **coding categories** | | **skip** |
| **C01** | | How old were you when someone told you you were HIV positive? | \| age in years \|  \|  \| \| --- \| --- \| --- \| | |  |
| **C02** | | Who first told you that you had HIV?  circle all that apply | \|  \| **yes** \| **no** \| **dk** \| \| --- \| --- \| --- \| --- \| \| health care provider \| **1** \| **2** \| **8** \| \| social worker \| **1** \| **2** \| **8** \| \| peer counselor \| **1** \| **2** \| **8** \| \| biological mother \| **1** \| **2** \| **8** \| \| biological father \| **1** \| **2** \| **8** \| \| grandmother \| **1** \| **2** \| **8** \| \| other female relative \| **1** \| **2** \| **8** \| \| other male relative \| **1** \| **2** \| **8** \| \| Other, ________________ \| **1** \| **2** \| **8** \| | | |
| **C03** | | Did you suspect that you had HIV before someone told you? | yes  no  don’t know | **1**  **2**  **8** | **>>C05**  **>>C05** |
| **C04** | | If yes, how old were you when you first started thinking you had HIV? | \| age in years \|  \|  \| \| --- \| --- \| --- \| | |  |
| **C05** | | Have you told anyone other than health care workers that you are HIV positive? | yes  no  don’t know | **1**  **2**  **8** |  |
| **C06** | | Have you disclosed to any of the following people?  read all items aloud and circle yes or no for each one. for items that are not applicable (e.g. respondent does not have children), circle na | \|  \| **yes** \| **no** \| **na** \| **dk** \| \| --- \| --- \| --- \| --- \| --- \| \| sibling \| **1** \| **2** \| **6** \| **8** \| \| other relative / family member \| **1** \| **2** \| **6** \| **8** \| \| friend \| **1** \| **2** \| **6** \| **8** \| \| school teacher \| **1** \| **2** \| **6** \| **8** \| | | |
| **C07** | | How concerned are you that people whom you have not disclosed to will find out your HIV status? Are you very concerned, a little concerned, or not at all concerned? | very concerned  a little concerned  not at all concerned  don't know | **1**  **2**  **3**  **8** |  |

| **D. SELF-EFFICACY (HIV Adherence and Self Efficacy Scale)** | | |
| --- | --- | --- |
| ***Read***: I am going to ask you about situations that could occur during your treatment for HIV. Treatment can involve different things for different people. Sometimes, this might refer to taking medications, and other times it could refer to other things that you do to deal with HIV such as diet and exercise or taking vitamins. So, in these questions, when I ask you about your ‘‘treatment’’ or your ‘‘treatment plan,’’ I am talking not only about any medications that you might be taking for HIV, but also other things that make up your self-care. For the following questions I will ask you to tell me in the past month, including today, how confident you have been that you can do the following things. Use this response scale ranging from 0 (‘‘cannot do at all’’) to 10 (‘‘completely certain can do’’). | | |
| **NO** | **QUESTIONS AND FILTERS** | **CODING CATEGORIES** |
| **D01** | In the past month, how confident have you been that you can stick to your treatment plan even when side effects begin to interfere with daily activities? | **00 (cannot do it at all – no confidence)**  **1**  **2**  **3**  **4**  **5 (neutral—may can do, maybe cannot**  **6**  **7**  **8**  **9**  **10 (complete certain can do – full confidence)** |
| **D02** | In the past month, how confident have you been that you can integrate your treatment into your daily routine? | **00 (cannot do it at all – no confidence)**  **1**  **2**  **3**  **4**  **5 (neutral—may can do, maybe cannot**  **6**  **7**  **8**  **9**  **10 (complete certain can do – full confidence)** |
| **D03** | In the past month, how confident have you been that you can integrate your treatment into your daily routine even if it means taking medication or doing other things in front of people who don’t know you are HIV-infected? | **00 (cannot do it at all – no confidence)**  **1**  **2**  **3**  **4**  **5 (neutral—may can do, maybe cannot**  **6**  **7**  **8**  **9**  **10 (complete certain can do – full confidence)** |
| **D04** | In the past month, how confident have you been that you can stick to your treatment schedule even when your daily routine is disrupted? | **00 (cannot do it at all – no confidence)**  **1**  **2**  **3**  **4**  **5 (neutral—may can do, maybe cannot**  **6**  **7**  **8**  **9**  **10 (complete certain can do – full confidence)** |
| **D05** | In the past month, how confident have you been that you can stick to your treatment schedule when you aren’t feeling well? | **00 (cannot do it at all – no confidence)**  **1**  **2**  **3**  **4**  **5 (neutral—may can do, maybe cannot**  **6**  **7**  **8**  **9**  **10 (complete certain can do – full confidence)** |
| **D06** | In the past month, how confident have you been that you can stick to your treatment schedule when it means changing your eating habits? | **00 (cannot do it at all – no confidence)**  **1**  **2**  **3**  **4**  **5 (neutral—may can do, maybe cannot**  **6**  **7**  **8**  **9**  **10 (complete certain can do – full confidence)** |
| **D07** | In the past month, how confident have you been that you can continue with your treatment even if doing so interferes with your daily activities? | **00 (cannot do it at all – no confidence)**  **1**  **2**  **3**  **4**  **5 (neutral—may can do, maybe cannot**  **6**  **7**  **8**  **9**  **10 (complete certain can do – full confidence)** |
| **D08** | In the past month, how confident have you been that you can continue with the treatment plan your clinician prescribed even if you are told your HIV is virally suppressed or HVL is undetectable? | **00 (cannot do it at all – no confidence)**  **1**  **2**  **3**  **4**  **5 (neutral—may can do, maybe cannot**  **6**  **7**  **8**  **9**  **10 (complete certain can do – full confidence)** |
| **D09** | In the past month, how confident have you been that you can continue with your treatment even when you are feeling discouraged about your health? | **00 (cannot do it at all – no confidence)**  **1**  **2**  **3**  **4**  **5 (neutral—may can do, maybe cannot**  **6**  **7**  **8**  **9**  **10 (complete certain can do – full confidence)** |
| **D10** | In the past month, how confident have you been that you can continue with your treatment even when getting to your clinic appointments is a major hassle? | **00 (cannot do it at all – no confidence)**  **1**  **2**  **3**  **4**  **5 (neutral—may can do, maybe cannot**  **6**  **7**  **8**  **9**  **10 (complete certain can do – full confidence)** |
| **D11** | In the past month, how confident have you been that you can continue with your treatment even when people close to you tell you that they don’t think that it Is doing any good? | **00 (cannot do it at all – no confidence)**  **1**  **2**  **3**  **4**  **5 (neutral—may can do, maybe cannot**  **6**  **7**  **8**  **9**  **10 (complete certain can do – full confidence)** |
| **D12** | In the past month, how confident have you been that you can get something positive out of your participation in treatment, even if the medication you are taking does not improve your health? | **00 (cannot do it at all – no confidence)**  **1**  **2**  **3**  **4**  **5 (neutral—may can do, maybe cannot**  **6**  **7**  **8**  **9**  **10 (complete certain can do – full confidence)** |

| **E. SELF-ESTEEM (Rosenberg Self-Esteem Scale)** |
| --- |
| **Read**: Now I will read a list of statements, and I want you to tell me what you think or feel about them, and how they relate to the thoughts and feelings that you have had. |
| Select one space in the interview area to represent that the respondent “Agrees” with the statement and a different space in the interview area to represent that the respondent “Disagrees" with the statement. After each statement, if the respondent moves to the “Agree” location, ask the respondent if s/he strongly agrees or just agrees. If the respondent moves to the “Disagree” location, ask the respondent if s/he disagrees or strongly disagrees. |
| **Read**: : If you agree that the statement represents your thoughts and feelings, then I would like you to hold up the GREEN card. If you disagree with the statement, then I would like you to hold up the RED card.  First, we are going to practice responding to these types of questions. As an example, I will read a statement that people your age sometimes say, think, or feel. I want you to show me whether you agree or disagree by moving to the designated spaces. |
| Wait for respondent move to hold up the card that represents her/his feelings. |
| [IF GREEN]: Now I would like to do know: do you like music and dance, or do you really like music and dance?  [IF RED]: Now I would like to know: do you dislike music and dance or do you really dislike music and dance? |
| If you feel comfortable that the respondent knows how to respond to the questions, continue. Otherwise, try another example “I like sports.” |
| [IF GREEN]: Now I would like to do know: do you just agree that [READ STATEMENT] or do you strongly agree?  [IF RED]: Now I would like to know: do you just disagree that [READ STATEMENT] or do you strongly disagree? |
| I may say that young people of your age think or say “I like music and dance.”  If you like music and dance, hold up the GREEN card  If you don’t like music and dance, hold up the RED card  **Read**: Now I will read a list of statements dealing with general feelings about yourself. Please show me how these statements describe your feelings by holding up the correct card, as we just did. Remind respondent the cards for “Agree” and “Disagree”. Remember to prompt whether the respondent agrees(disagrees) or strongly agrees(disagrees) when the respondent pics up the appropriate card. Record the appropriate code.  [IF GREEN]: Now I would like to do know: do you just agree that [READ STATEMENT] or do you strongly agree?  [IF RED]: Now I would like to know: do you just disagree that [READ STATEMENT] or do you strongly disagree? |

| **NO** | **QUESTIONS AND FILTERS** | **CODING CATEGORITES** | | |
| --- | --- | --- | --- | --- |
| **E01** | On the whole, I am satisfied with myself | STRONGLY AGREE  AGREE  DISAGREE  STRONGLY DISAGREE | **01**  **02**  **03**  **04** |  |
| **E02** | At times I think I am no good at all | STRONGLY AGREE  AGREE  DISAGREE  STRONGLY DISAGREE | **01**  **02**  **03**  **04** |  |
| **E03** | I feel that I have a number of good qualities | STRONGLY AGREE  AGREE  DISAGREE  STRONGLY DISAGREE | **01**  **02**  **03**  **04** |  |
| **E04** | I am able to do things as well as most other people | STRONGLY AGREE  AGREE  DISAGREE  STRONGLY DISAGREE | **01**  **02**  **03**  **04** |  |
| **E05** | I feel I do not have much to be proud of | STRONGLY AGREE  AGREE  DISAGREE  STRONGLY DISAGREE | **01**  **02**  **03**  **04** |  |
| **E06** | I certainly feel useless at times | STRONGLY AGREE  AGREE  DISAGREE  STRONGLY DISAGREE | **01**  **02**  **03**  **04** |  |
| **E07** | I feel that I’m a person of worth, at least on an equal plane with others | STRONGLY AGREE  AGREE  DISAGREE  STRONGLY DISAGREE | **01**  **02**  **03**  **04** |  |
| **E08** | I wish I could have more respect for myself | STRONGLY AGREE  AGREE  DISAGREE  STRONGLY DISAGREE | **01**  **02**  **03**  **04** |  |
| **E09** | All in all, I am inclined to feel that I am a failure | STRONGLY AGREE  AGREE  DISAGREE  STRONGLY DISAGREE | **01**  **02**  **03**  **04** |  |
| **E10** | I take a positive attitude toward myself | STRONGLY AGREE  AGREE  DISAGREE  STRONGLY DISAGREE | **01**  **02**  **03**  **04** |  |

| 1. **GENERAL HEALTH QUESTIONNAIRE (GHQ-12)** | | | | |
| --- | --- | --- | --- | --- |
| **NO** | **QUESTIONS AND FILTERS** | **CODING CATEGORIES** | | |
| **F01** | During the past two weeks, have you been able to concentrate on whatever you are doing? | LESS THAN USUAL  NO MORE THAN USUAL  RATHER MORE THAN USUAL  MUCH MORE THAN USUAL | **0**  **1**  **2**  **3** |  |
| **F02** | During the past two weeks, have you lost much sleep over worry? | LESS THAN USUAL  NO MORE THAN USUAL  RATHER MORE THAN USUAL  MUCH MORE THAN USUAL | **0**  **1**  **2**  **3** |  |
| **F03** | During the past two weeks, have you felt that you were playing a useful part in things? | LESS THAN USUAL  NO MORE THAN USUAL  RATHER MORE THAN USUAL  MUCH MORE THAN USUAL | **0**  **1**  **2**  **3** |  |
| **F04** | During the past two weeks, have you felt capable about making decisions about things? | LESS THAN USUAL  NO MORE THAN USUAL  RATHER MORE THAN USUAL  MUCH MORE THAN USUAL | **0**  **1**  **2**  **3** |  |
| **F05** | During the past two weeks, have you felt constantly under strain? | LESS THAN USUAL  NO MORE THAN USUAL  RATHER MORE THAN USUAL  MUCH MORE THAN USUAL | **0**  **1**  **2**  **3** |  |
| **F06** | During the past two weeks, have you felt that you couldn’t overcome your difficulties? | LESS THAN USUAL  NO MORE THAN USUAL  RATHER MORE THAN USUAL  MUCH MORE THAN USUAL | **0**  **1**  **2**  **3** |  |
| **F07** | During the past two weeks, have you been able to enjoy your normal day-to-day activities? | LESS THAN USUAL  NO MORE THAN USUAL  RATHER MORE THAN USUAL  MUCH MORE THAN USUAL | **0**  **1**  **2**  **3** |  |
| **F08** | During the past two weeks, have you been able ot face up to your problems? | LESS THAN USUAL  NO MORE THAN USUAL  RATHER MORE THAN USUAL  MUCH MORE THAN USUAL | **0**  **1**  **2**  **3** |  |
| **F09** | During the past two weeks, have you been feeling unhappy and depressed? | LESS THAN USUAL  NO MORE THAN USUAL  RATHER MORE THAN USUAL  MUCH MORE THAN USUAL | **0**  **1**  **2**  **3** |  |
| **F10** | During the past two weeks, have you been losing confidence in yourself? | LESS THAN USUAL  NO MORE THAN USUAL  RATHER MORE THAN USUAL  MUCH MORE THAN USUAL | **0**  **1**  **2**  **3** |  |
| **F11** | During the past two weeks, have you been thinking of yourself as a worthless person? | LESS THAN USUAL  NO MORE THAN USUAL  RATHER MORE THAN USUAL  MUCH MORE THAN USUAL | **0**  **1**  **2**  **3** |  |
| **F12** | During the past two weeks, have you been feeling reasonably happy, all things considered? | LESS THAN USUAL  NO MORE THAN USUAL  RATHER MORE THAN USUAL  MUCH MORE THAN USUAL | **0**  **1**  **2**  **3** |  |

| **G. OTHER TYPES OF CARE & SUPPORT** | | | | |
| --- | --- | --- | --- | --- |
| **no** | **questions and filters** | **coding categories** | | |
| **G01** | Have you ever attended a group with other children or young people who are also living with HIV to talk about your experiences? | yes  no  don’t know | **1**  **2**  **8** |  |
| **G02** | What was this group called? | \|  \| **yes** \| **no** \| **na** \| **dk** \| \| --- \| --- \| --- \| --- \| --- \| \| Ariel club \| **1** \| **2** \| **6** \| **8** \| \| OTHER \| **1** \| **2** \| **6** \| **8** \|   SPECIFY_________________________________ | | |
| **G03** | Where did you last attend such a group? | CTC  community location  other ______________________________ |  |  |
| **G04** | How old were you when you first attended such a group? | \| age in years \|  \|  \| \| --- \| --- \| --- \| | |  |
| **G05** | About how often have you attended such a group in the past one year? | seldom, only once or twice  sometimes, about half of the times they happen  often, almost all the times they happen  don’t know | **1**  **2**  **3**  **8** |  |
| **G06** | Does the group you attend have an age limit, so that people reaching this age graduate from that group (and stop attending)? | yes  no  don’t know | **1**  **2**  **8** |  |
| **G07** | Is there someone you can turn to for advice or encouragement when you feel disappointed or confused? | yes  no  don’t know | **1**  **2**  **8** |  |

| **G08** | There is someone I can talk to if I am upset, nervous or depressed. | **strong disagree** | **disagree** | **agree** | **strong agree** | **NA** |
| --- | --- | --- | --- | --- | --- | --- |
| **G09** | There is someone I can contact if I want to talk about an important personal problem. | **strong disagree** | **disagree** | **agree** | **strong agree** | **NA** |
| **G010** | My neighbors trust me. | **strong disagree** | **disagree** | **agree** | **strong agree** | **NA** |
| **G011** | I trust my neighbors. | **strong disagree** | **disagree** | **agree** | **strong agree** | **NA** |
| **G012** | There is someone I can turn to if I needed to borrow money, get help getting to the doctor, or some other small immediate help. | **strong disagree** | **disagree** | **agree** | **strong agree** | **NA** |
| **G013** | The people in my personal life give me information, suggestions, or guidance when I need it. | **strong disagree** | **disagree** | **agree** | **strong agree** | **NA** |
| **G014** | My neighbors are comfortable talking to me about their problems. | **strong disagree** | **disagree** | **agree** | **strong agree** | **NA** |

| 1. **Conclusion** | | | | |
| --- | --- | --- | --- | --- |
| **NO** | **QUESTIONS AND FILTERS** | **CODING CATEGORIES** | | **SKIP** |
| **H01** | Did the respondent terminate the survey early? | YES  NO | **1**  **2** | **>> Q2** |
| **H01A** | Why did the respondent terminate the survey early? | TEMPORARY STOP ONLY. WISHES TO CONTINUE SURVEY AT A LATER TIME  TIRED  TOO BUSY / DOESN’T HAVE TIME  OFFENDED AT QUESTION  SUSPICIOUS OF ENUMERATOR OR SURVEY INTENT  DOES NOT FEEL LIKE CONTINUING SURVEY  OTHER ­­­­­­____________________________  DON’T KNOW | **1**  **2**  **3**  **4**  **5**  **6**  **7**  **8** |  |
| **H02** | Were any other survey enumeration team staff present during the interview? | YES  NO | **1**  **2** | **>> Q3** |
| **H02A** | Please record the ID numbers of all staff other than the enumerator who were present for the interview | **ID NUMBERS___________________________________**  **_____________________________________________** | |  |
| **H03** | Were any other individuals present for the interview? | YES  NO | **1**  **2** | **>> Q4** |
| **H03A** | What is their relationship to the respondent? | **RELATIONSHIP__________________________________**  **______________________________________________** | |  |
| **H04** | Are you very confident, somewhat confident or not very confident in the overall quality and truthfulness of this respondent’s responses? | VERY CONFIDENT  SOMEWHAT CONFIDENT  NOT CONFIDENT | **1**  **2**  **3** | **>> End** |
| **H04A** | Why are you not confident? | **SPECIFY___________________________________**  ___________________________________________________ |  |  |

| Interview end time: |  |  |  | : |  |  |  |
| --- | --- | --- | --- | --- | --- | --- | --- |
|  | | hour | | minute | | | |
