# Supplementary material for: Adolescent support club attendance and self-efficacy associated with HIV treatment outcomes in Tanzania
Source: PLOS Glob Public Health. 2022 Oct 3;2(10):e0000065. doi: 10.1371/journal.pgph.0000065 (PMC10021176; doi:10.1371/journal.pgph.0000065)
Supplement: S2 Text — (DOCX) [file pgph.0000065.s002.docx]

**Dodoso kwa ajili ya Vijana-balehe**

|  |  |  | **ME 1**  **KE 2** |
| --- | --- | --- | --- |
| **TAREHE** | **ALAMA MAALUM YA RA** | **KITAMBULISHO CHA MGONJWA (PTID)** | **JINSI** |
|  |  |  |  |

| Muda wa kuanza mahojiano: |  |  |  | : |  |  |  |
| --- | --- | --- | --- | --- | --- | --- | --- |
|  | | saa | | dakika | | | |

| **A. TAARIFA ZA AWALI KUHUSU KAYA NA IDADI YA WATU** | | | | | | |
| --- | --- | --- | --- | --- | --- | --- |
| **na** | **maswali na vigezo vya kuchambua majibu** | | **alama maalum za majibu ya maswali** | | | **ruka** |
| **A01** | Unaishi katika mkoa gani hivi sasa? | | arusha  kilimanjaro | | **1**  **2** |  |
| **A02** | Je, ni kwa muda gani umeishi mahali hapa? | | \| miaka \|  \|  \| \| --- \| --- \| --- \| \| iwapo ni < mwaka1 \| **00** \| \| | | |  |
| **A03** | Pamoja na wewe mwenyewe, kuna watu wangapi wanaoishi ndani ya kaya yako hivi sasa? | | \| idadi ya watu \|  \|  \| \| --- \| --- \| --- \| | | |  |
| **A04** | Ni nani mlezi wako mkuu (primary guardian)? | | Mzazi  Bibi/Babu  Ndugu mwingine mtu mzima (dada, shangazi/mama mdogo, mjomba/ baba mkubwa au mdogo, n.k.)  Mtu mwingien ambae siyo ndugu (jirani, rafiki, mwanajamii)  MWINGINE, eleza ____________________ | | **1**  **2**  **3**  **4**  **5**  **6** |  |
| **A05** | Je, mlezi wako mkuu ni wa jinsi gani ? | | Me  Ke | | **1**  **2** |  |
| **A06** | Je, wewe au mkazi mwingine katika kaya yako mnamiliki …  soma chaguzi zote kisha zungushia jibu linalohusika kwa kila chaguzi | \|  \| **ndy** \| **hpn** \| **sf** \| \| --- \| --- \| --- \| --- \| \| redio \| **1** \| **2** \| **8** \| \| runinga (tv) \| **1** \| **2** \| **8** \| \| simu ya mkononi \| **1** \| **2** \| **8** \| \| jokofu (friji) \| **1** \| **2** \| **8** \| \| baiskeli \| **1** \| **2** \| **8** \| \| pikipiki \| **1** \| **2** \| **8** \| \| mkokoteni wa kusukuma na mnyama \| **1** \| **2** \| **8** \| \| gari/lori \| **1** \| **2** \| **8** \| | | | |  |
| **A07** | Je ni chanzo gani kikuu cha mwanga kwa ajili ya taa ndani ya kaya yako? | | Umeme wa kawaida (TANESCO)  Umeme wa kutumia mionzi ya jua (sola)  Gesi  Taa ya mafuta  Nyingine | **1**  **2**  **3**  **4**  **7** | |  |
| **A08** | Ni nini chanzo kikuu cha maji ya kunywa kwa ajili ya wanakaya? | | Bomba  Kisima cha kuchimba  Chemchem  Maji ya kununua ya chupa (bottled water)  Nyingine | **1**  **2**  **3**  **4**  **9** | |  |
| **A09** | Je, ni aina gani ya choo ambacho wanakaya unaoishi nao wanatumia? | | Cha kuflashi  Cha shimo  Nyingine | **1**  **2**  **7** | |  |
| **A10** | Kaya yako inatumia nishati gani kupikia chakula? | | Umeme  Gesi ya mtungi  Mafuta ya taa  Mkaa  Kuni  Nyingine | **1**  **2**  **3**  **4**  **5**  **8** | |  |
| **A11** | Nyumba yako ina sakafu ya aina gani? | | Udongo  Saruji/Sementi  Malumalu  Nyingine | **1**  **2**  **3**  **4** | |  |

| **A12** | Kuta za nyumba yako zimetengenezwa na nini? | Saruji/Sementi  Matofali ya kuchoma  Matofali mabichi  Udongo/Makuti  Nyingine___________________________ | **1**  **2**  **3**  **4**  **5** |  |
| --- | --- | --- | --- | --- |
| **A13** | Paa la nyumba yako ni la ina gani? | Bati /Vigae  Nyasi  Nyingine_______________________ | **1**  **2**  **3** |  |
| **A14** | Unaweza kuniambia familia yako inamiliki ng’ombe wangapi? | <5  5-9  10-19  20+ | **1**  **2**  **3**  **4** |  |
| **A15** | Unaweza kuniambia familia yako inamiliki kondoo/ mbuzi wangapi? | <5  5-9  10-19  20+ | **1**  **2**  **3**  **4** |  |
| **A16** | Katika miezi sita iliyopita, ni mara nyingi kiasi gani kaya yako ilishindwa kupata mahitaji yake ya chakula? Unaweza kusema ‘hata mara moja’, mara chache, wakati mwingine, mara nyingi au kila wakati? | hata mara moja  mara chache  wakati mwingine  mara nyingi  kila wakati | **1**  **2**  **3**  **4**  **5** |  |

| **A17** | Una umri gani (miaka kamili)?  jaza mwaka wa kuzaliwa au umri, lakini siyo vyote viwili. dadisi ili kuweza kukadiria umri wa mshiriki (angalau kwa miaka 5 ya umri halisi) iwapo mshiriki hataweza kutaja umri au mwaka wa kuzaliwa | \| umri kwa miaka \|  \|  \| \| --- \| --- \| --- \| | |  |
| --- | --- | --- | --- | --- | --- | --- | --- |
| **A18** | Je, unakiwango gani cha elimu ulichofikia? | hajasoma  miaka kadhaa ya elimu ya msingi  Elimu ya msingi  Miaka kadhaa ya sekondari  Elimu ya sekondari  Elimu ya juu (Chuo, Chuo Kikuu na zaidi) | **1**  **2**  **3**  **4**  **5**  **6** |  |
| **A19** | Je, umewahi kuolewa au kuoa? | Ndiyo, bado niko katika ndoa  Ndiyo, Nimetaliki/Talikiwa  Ndiyo, Nimefiwa na mwenzi  Hapana | **1**  **2**  **3**  **4** | **>>A21** |
| **A20** | Iwapo jibu ni ‘ndiyo’, ulikuwa na umri gani ulipoolewa/kuoa kwa mara ya kwanza? | umri kwa miaka   \|  \|  \| \| --- \| --- \| |  | **>>A22** |

| **A21** | Je, umewahi kuwa na uhusiano wa kimapenzi? | Ndiyo  Hapana | **1**  **2** |  |
| --- | --- | --- | --- | --- |
| **A22** | Umewahi kufanya mapenzi (kujamiana)? | Ndiyo  Hapana | **1**  **2** |  |
| **A23** | Iwapo jibu ni ‘ndiyo’, ulikuwa na umri gani ulipofanya mapenzi kwa mara ya kwanza? | umri kwa miaka   \|  \|  \| \| --- \| --- \| |  |  |
| **A24** | Tofauti ya umri kati yako na mpenzi wako wa kwanza ilikuwa ni miaka mingapi? | chini ya miaka 2  miaka 2-4  miaka 5-9  miaka 10+ | **1**  **2**  **3**  **4** |  |
| **A25** | Je, umewahi kufanya mapenzi bila ridhaa yako? Yaani, kufanya mapenzi na mtu ambae alikushinikiza au kukulazimisha ulipotaka kukataa? | Ndiyo  Hapana | **1**  **2** |  |
| **A26** | Je, umewahi kushika ujauzito (mimba)? | Ndiyo  Hapana | **1**  **2** | **>>Ruka hadi sehemu inayofuata** |
| **A27** | Iwapo jibu ni ‘ndiyo’, ulikuwa na umri gani uliposhika ujauzito wa mara ya kwanza? | umri kwa miaka   \|  \|  \| \| --- \| --- \| |  |  |

| **B. UFAHAMU KUHUSU TIBA NA MATUNZO YA VVU** | | | | |
| --- | --- | --- | --- | --- |
| **na** | **maswali na vigezo vya kuchambua majibu** | **alama maalum za majibu ya maswali** | | **ruka** |
| **B01** | Ulikuwa na umri gani ulipoanza kupata tiba na matunzo ya VVU kwa mara ya kwanza? | \| umri kwa miaka \|  \|  \| \| --- \| --- \| --- \| | |  |
| **B02** | Ni kwa muda gani umekua ukipata huduma kutoka kwenye kliniki hii ya tiba ya VVU na matunzo kwa muda gani? | \| miezi \|  \|  \| \| --- \| --- \| --- \| \| miaka \|  \|  \| \| sifahamu \| **88** \| \| | |  |
| **B03** | Umewahi kupata huduma kutoka kwenye kliniki ngapi tofauti kabla ya kituo hiki? | \| Idadi \|  \|  \| \| --- \| --- \| --- \| | |  |
| **B04** | Je, umeshaanza kutumia dawa za ARV? | ndiyo  hapana  sifahamu | **1**  **2**  **8** |  |
| **B05** | Ulianza lini kutumia dawa za ARV?  Jaza miezi, miaka iliyopita | \| miezi iliyopita \|  \|  \| \| --- \| --- \| --- \| \| miaka iliyopata \|  \|  \| \| sifahamu \| **88** \| \| | |  |
| **B06** | Ulikuwa na umri gani ulipoanza kutumia dawa za ARV kwa mara ya kwanza? | \| umri kwa miaka \|  \|  \| \| --- \| --- \| --- \| | |  |
| **B07** | Je, bado unaendelea kutumia dawa za ARV? | ndiyo  hapana  sifahamu | **1**  **2**  **8** |  |
| **B08** | Katika siku 7 zilizopita, ni mara ngapi ulishindwa kumeza dawa moja au zaidi ya moja ya ARV? | idadi ya siku ambazo dawa moja au zaidi hazikumezwa: _____  Sifahamu | **8** |  |
| **B09** | Baadhi ya watu wanaona kwamba wanaweza wasimeze dawa kwa siku 2 au zaidi mfulululizo. Umewahi kushindwa/kuruka kumeza dawa zako ZOTE kwa siku 2 au zaidi mfululizo? | ndiyo  hapana  sifahamu | **1**  **2**  **8** |  |
| **B10** | Je, unae mtu wa kukusaidia kumeza dawa? (treatment supporter), yaani, mtu katika kaya yako ambae anasaidia kukukumbusha umeze dawa na kuhudhuria kliniki? | ndiyo  hapana  sifahamu | **1**  **2**  **8** |  |
| **B11** | Je, mlezi/mtu anayekusaidia kumeza dawa wanahudhuria kliniki ya tiba ya VVU kwa ajili ya huduma za tiba ya VVU kwa ajili yao wao wenyewe? | ndiyo  hapana, hawana maambukizi ya VVU  hapana, wana maambukizi ya VVU lakini hawahudhurii kliniki  sifahamu | **1**  **2**  **3**  **8** |  |
| **B12** | Je, mlezi/mtu wa kukusaidia kumeza dawa anakusindikiza kliniki au unakwenda pekee yako? | Nakwenda pekee yangu  naenda na mlezi/ Msaidizi wa matibabu  Nyingine | **1**  **2**  **3** |  |
| **B13** | Unatumia usafiri wa aina gani unapoenda kliniki? | Natembea  Teksi ya basikeli (ya kukodi)  Boda boda  Dala Dala  Teksi  Baiskeli binafsi/ya kuazima  Gari binafsi  Nyingine (eleza)__________ | 1  2  3  4  5  6  7  8 |  |
| **B14** | Ulipofika kliniki mara ya mwisho ulitumia kiasi gani kwa ajili ya usafiri?  hesabu gharama ya kwenda au kurudi tu (one way). iwapo gharama ya kwenda na kurudi inatofautiana chagua iliyo juu zaidi | \| Shilingi za Kitanzania \|  \| \| --- \| --- \| \| sifahamu \| **888 888** \| \| Amekataa kujibu \| **999 999** \| | | |
| **B15** | Ilikuchukua muda gani kutoka nyumbani hadi kliniki ulipoenda kliniki mara ya mwisho | \| Saa:dakika \|  \|  \| **:** \|  \|  \| \| --- \| --- \| --- \| --- \| --- \| --- \| \|  \|  \| \| \| \| \| | | |
| **B16** | Kwa ujumla, mara zote ulizoenda kliniki, inakuchukua muda gani kupata huduma zote kuanzia unapofika hadi unapomaliza? | Chini ya saa 1  saa 1 - 3  masaa 4 - 6  zaidi ya masaa 6  sifahamu  amekataa kujibu | **1**  **2**  **3**  **4**  **8**  **9** |  |
| **B17** | Je, mtu mwenye VVU anapoanza kutumia dawa za VVU, uwezekano wa yeye kumwambukiza mwenzi wake unaongezeka, unapungua au unabakia vile vile? | inaongezeka  inapungua  inabaki vilevile  sifahamu | **1**  **2**  **8** |  |
| **B18** | Je, kuwa na CD4 chache ni vizuri au vibaya kwa afya yako? | vizuri  vibaya  sifahamu | **1**  **2**  **8** |  |
| **B19** | Mwanamke mjamzito mwenye VVU anaweza kumwambukiza mtoto wake ambae hajazaliwa? | ndiyo  hapana  sifahamu | **1**  **2**  **8** |  |
| **B20** | Mwanamke mwenye VVU anaweza kumwambukiza mtoto wake kupitia maziwa wakati wa kumnyonyesha? | ndiyo  hapana  sifahamu | **1**  **2**  **8** |  |
| **B21** | Unaweza kupata VVU ukifanya mapenzi (ngono) na mtu anaeonekana mwenye afya nzuri? | ndiyo  hapana  sifahamu | **1**  **2**  **8** |  |
| **B22** | UKIMWI una tiba? | ndiyo  hapana  sifahamu | **1**  **2**  **8** |  |

| **C. Kuwaambia watu wengine kuhusu hali ya maambukizi** | | | | | |
| --- | --- | --- | --- | --- | --- |
| **na** | **maswali na vigezo vya kuchambua majibu** | | **alama maalum za majibu ya maswali** | | **ruka** |
| **C01** | | Ulikuwa na umri gani ulipoambiwa kwamba unaishi na VVU? | \| umri kwa miaka \|  \|  \| \| --- \| --- \| --- \| | |  |
| **C02** | | Nani alikuwa wa kwanza kukuambia kwamba unaishi na VVU?  zungushia majibu yote yanayohusika | \|  \| **ndy** \| **hpn** \| **sf** \| \| --- \| --- \| --- \| --- \| \| mhudumu wa afya \| **1** \| **2** \| **8** \| \| afisa wa jamii \| **1** \| **2** \| **8** \| \| mwelimishaji rika \| **1** \| **2** \| **8** \| \| mama mzazi \| **1** \| **2** \| **8** \| \| baba mzazi \| **1** \| **2** \| **8** \| \| bibi/babu \| **1** \| **2** \| **8** \| \| ndugu mwingine wa kike \| **1** \| **2** \| **8** \| \| ndugu mwingine wa kiume \| **1** \| **2** \| **8** \| \| Nyingine_______________ \| **1** \| **2** \| **8** \| | | |
| **C03** | | Je, ulihisi kwamba una maambukizi ya VVU kabla hujaelezwa na mtu? | ndiyo  hapana  sifahamu | **1**  **2**  **8** | **>>C05**  **>>C05** |
| **C04** | | Iwapo jibu ni ‘ndiyo’, ulikuwa na umri gani ulipoanza kuhisi kwamba una maambukizi ya VVU? | \| umri kwa miaka \|  \|  \| \| --- \| --- \| --- \| | |  |
| **C05** | | Umeshamwambia mtu mwingine yeyote mbali na wahudumu wa afya kwamba una maambukizi ya VVU? | ndiyo  hapana  sifahamu | **1**  **2**  **8** |  |
| **C06** | | Je, umeshawaeleza yeyote kati ya watu wafuatao kuhusu hali yako ya maambukizi?  soma chaguzi zote za majibu kwa sauti kisha zungushia ‘ndy’ au ‘hpn’ kwa kila chaguzi la jibu. kwa majibu ambayo hayahusiki (k.m. mshiriki hana watoto), zungushia ‘hsk’ | \|  \| **ndy** \| **hpn** \| **hsk** \| **sf** \| \| --- \| --- \| --- \| --- \| --- \| \| ndugu wa kuzaliwa \| **1** \| **2** \| **6** \| **8** \| \| ndugu mwingine / mwanafamilia \| **1** \| **2** \| **6** \| **8** \| \| rafiki \| **1** \| **2** \| **6** \| **8** \| \| mwalimu wa shule \| **1** \| **2** \| **6** \| **8** \| | | |
| **C07** | | Unajali kiasi gani kwamba watu ambao hujawaeleza watafahamu kuhusu hali yako ya maambukizi ya VVU? Je, unajali sana, unajali kidogom hujali hata kidogo? | najali sana  najali kidogo  sijali hata kidogo  sifahamu | **1**  **2**  **3**  **8** |  |

| **D. UFANISI BINAFSI (SELF-EFFICACY) (Ufuataji wa masharti ya matibabu ya VVU na kipimo cha ufanisi binafsi)** | | |
| --- | --- | --- |
| ***Soma***: Nitakuuliza kuhusu hali ambazo zinaweza kutokea katika kipindi cha matibabu unayopata kwa ajili ya VVU. Matibabu yanaweza kuhusisha mambo tofauti kwa watu tofuati. Wakati mwingine, yanaweza yakahusu matumizi ya dawa, na wakati mwingine yanaweza yakahusu mambo mengine ambayo unayafanya kukabiliana na hali ya maambukizi ya VVU kama vile lishe na mazoezi au kumeza dawa za kuongeza vitamini. Kwahiyo, katika haya maswali, ninapokuuliza kuhusu “matibabu unayopata” au “mpango wa matibabu” ninazungumzia siyo tu matumizi ya dawa kwa ajili ya VVU, bali pia mambo mengine yanayohusu jinsi unavyojitunza wewe mwenyewe (self-care). Kwa maswali yanayofuata nitakuomba unieleze katika mwezi uliopita, ukijumlisha siku ya leo, ulijisikia kujiamini kwa kiasi gani kwamba unaweza kufanya mambo yafuatayo. Tumia mizani ifuatayo ya majibu kuanzia 0 (‘‘siwezi kabisa’’) hadi 10 (‘‘nina uhakika kwamba naweza’’). | | |
| **NA** | **MASWALI NA VIGEZO VYA KUCHUJIA MAJIBU** | **ALAMA MAALUM ZA MAJIBU YA MASWALI** |
| **D01** | Katika mwezi uliopita, ulikuwa unajiamini kiasi gani kwamba unaweza kufuata masharti ya mpango wa matibabu hata pale madhara ya dawa yatakapoingilia shughuli zako za kila siku? | **00 (siwezi kabisa – sijiamini)**  **1**  **2**  **3**  **4**  **5 (inawezekana nikaweza au nisiweze - najiamini kiasi)**  **6**  **7**  **8**  **9**  **10 (nina uhakika kwamba naweza – najiamini kabisa)** |
| **D02** | Katika mwezi uliopita, ulikuwa unajiamini kiasi gani kwamba unaweza kuingiza (kujumuisha) ratiba yako ya matibabu katika ratiba yako ya kila siku? | **00 (siwezi kabisa – sijiamini)**  **1**  **2**  **3**  **4**  **5 (katikati...inawezekana nikaweza au nisiweze - najiamini kiasi)**  **6**  **7**  **8**  **9**  **10 (nina uhakika kwamba naweza – najiamini kabisa)** |
| **D03** | Katika mwezi uliopita, ulikuwa unajiamini kiasi gani kwamba unaweza kuingiza (kujumuisha) ratiba yako ya matibabu katika ratiba yako ya kila siku hata ikibidi kumeza dawa au kufanya vitu vingine mbele za watu ambao hawafahamu kwamba una maambukizi ya VVU? | **00 (siwezi kabisa – sijiamini)**  **1**  **2**  **3**  **4**  **5 (katikati … inawezekana nikaweza au nisiweze - najiamini kiasi)**  **6**  **7**  **8**  **9**  **10 (nina uhakika kwamba naweza – najiamini kabisa)** |
| **D04** | Katika mwezi uliopita, ulikuwa unajiamini kiasi gani kwamba unaweza kufuata ratiba yako ya matibabu hata pale ambapo ratiba yako ya kila siku inapovurugika? | **00 (siwezi kabisa – sijiamini)**  **1**  **2**  **3**  **4**  **5 (inawezekana nikaweza au nisiweze - najiamini kiasi)**  **6**  **7**  **8**  **9**  **10 (nina uhakika kwamba naweza – najiamini kabisa)** |
| **D05** | Katika mwezi uliopita, ulikuwa unajiamini kiasi gani kwamba unaweza kufuata ratiba yako ya matibabu hata pale ambapo hujisikii vizuri (unaumwa)? | **00 (siwezi kabisa – sijiamini)**  **1**  **2**  **3**  **4**  **5 (katikati … inawezekana nikaweza au nisiweze - najiamini kiasi)**  **6**  **7**  **8**  **9**  **10 (nina uhakika kwamba naweza – najiamini kabisa)** |
| **D06** | Katika mwezi uliopita, ulikuwa unajiamini kiasi gani kwamba unaweza kufuata ratiba yako ya matibabu pale inapohitaji ubadilishe tabia za ulaji wa chakula (eating habits)? | **00 (siwezi kabisa – sijiamini)**  **1**  **2**  **3**  **4**  **5 (katikati … inawezekana nikaweza au nisiweze - najiamini kiasi)**  **6**  **7**  **8**  **9**  **10 (nina uhakika kwamba naweza – najiamini kabisa)** |
| **D07** | Katika mwezi uliopita, ulikuwa unajiamini kiasi gani kwamba unaweza kuendelea na matibabu yako hata kama kufanya hivyo kutaingilia shughuli zako za kila siku? | **00 (siwezi kabisa – sijiamini)**  **1**  **2**  **3**  **4**  **5 (katikati … inawezekana nikaweza au nisiweze - najiamini kiasi)**  **6**  **7**  **8**  **9**  **10 (nina uhakika kwamba naweza – najiamini kabisa)** |
| **D08** | Katika mwezi uliopita, ulikuwa unajiamini kiasi gani kwamba unaweza kuendelea na mpango wa matibabu ambao daktari wako amekuandikia hata kama ukiambiwa kwamba uzalishaji wa VVU mwilini umedhibitiwa au VVU ndani ya damu havionekani? | **00 (siwezi kabisa – sijiamini)**  **1**  **2**  **3**  **4**  **5 (inawezekana nikaweza au nisiweze - najiamini kiasi)**  **6**  **7**  **8**  **9**  **10 (nina uhakika kwamba naweza – najiamini kabisa)** |
| **D09** | Katika mwezi uliopita, ulikuwa unajiamini kiasi gani kwamba unaweza kuendelea na matibabu hata pale unapokuwa umekata tamaa kuhusu hali yako ya afya? | **00 (siwezi kabisa – sijiamini)**  **1**  **2**  **3**  **4**  **5 (katikati … inawezekana nikaweza au nisiweze - najiamini kiasi)**  **6**  **7**  **8**  **9**  **10 (nina uhakika kwamba naweza – najiamini kabisa)** |
| **D10** | Katika mwezi uliopita, ulikuwa unajiamini kiasi gani kwamba unaweza kuendelea na matibabu hata pale kufika kliniki tarehe uliyopangiwa ilikuwa ni changamoto? | **00 (siwezi kabisa – sijiamini)**  **1**  **2**  **3**  **4**  **5 (katikati … inawezekana nikaweza au nisiweze - najiamini kiasi)**  **6**  **7**  **8**  **9**  **10 (nina uhakika kwamba naweza – najiamini kabisa)** |
| **D11** | Katika mwezi uliopita, ulikuwa unajiamini kiasi gani kwamba unaweza kuendelea na matibabu hata pale ambapo watu wako wa karibu wanakuambia kwamba hawadhani kama matibabu unayopata yanasaidia? | **00 (siwezi kabisa – sijiamini)**  **1**  **2**  **3**  **4**  **5 (inawezekana nikaweza au nisiweze - najiamini kiasi)**  **6**  **7**  **8**  **9**  **10 (nina uhakika kwamba naweza – najiamini kabisa)** |
| **D12** | Katika mwezi uliopita, ulikuwa unajiamini kiasi gani kwamba unaweza kupata faida kutokana na matibabu unayopata hata pale ambapo dawa unazotumia hazisaidii kuboresha afya yako? | **00 (siwezi kabisa – sijiamini)**  **1**  **2**  **3**  **4**  **5 (inawezekana nikaweza au nisiweze - najiamini kiasi)**  **6**  **7**  **8**  **9**  **10 (nina uhakika kwamba naweza – najiamini kabisa)** |

| **E. KIWANGO CHA KUJITHAMINI (Mizani ya Rosenberg ya kiwango cha kujithamini)** |
| --- |
| **Soma:** Sasa nitasoma orodha ya sentensi, na kwa kila sentensi nataka uniambie una mawazo au hisia gani kuhusu sentensi hiyo, na jinsi inavyohusiana na mawazo au hisia ambazo umekuwa nazo. |
| **Maelezo kwa Mhojaji:** Katika kipengele hiki, mshiriki anatakiwa kuonyesha iwapo anakubaliana au hakubaliani na sentensi ambazo zimeorodheshwa hapo chini. Baada ya kuonyesha msimamo wake wa kukubaliana au kutokukubaliana na sentensi, atatakiwa kueleza kiwango ambacho anakubaliana au hakubaliani na kila sentensi katika orodha. |
| **Soma:** Kwanza, tutafanya majaribio kuhusu namna ya kujibu maswali ya aina hii. Kama mfano, nitasoma sentensi yenye maneno, mawazo au hisia za vijana wa umri wako. Nataka unionyeshe iwapo unakubaliana au hukubaliani na sentensi nitakayosoma kwa kuinua kadi ya KIJANI kama unakubaliana na sentensi au kadi NYEKUNDU iwapo hukubaliani na sentensi.  **Soma** sentensi **ya mfano: “***Napenda muziki na kucheza dansi*!”  Iwapo unapenda muziki na kucheza dansi, inua kadi ya KIJANI  Iwapo hupendi muziki na kucheza dansi, inua kadi NYEKUNDU |
| **Subiri mshiriki achague kadi inayoendana na msimamo wake aidha wa kukubaliana au kutokukubaliana na** sentensi **uliyoisoma.** |
| **Soma:** Sasa nitakuuliza maswali ya ziada kuelewa kiwango chako cha kukubaliana au kutokukubaliana na sentensi husika.    **[Iwapo amechagua kadi ya KIJANI]**: Sasa nataka nifahamu: Unapenda muziki na kucheza dansi, au unapenda sana muziki na kucheza dansi?  **[Iwapo amechagua kadi NYEKUNDU]**: Sasa nataka nifahamu: Hupendi muziki na kucheza dansi, au hupendi muziki wala kucheza dansi hata kidogo? |
| **Iwapo unaona kwamba mshiriki ameelewa jinsi ya kujibu maswali, endelea kumsomea** sentensi **zilizoorodheshwa hapo chini. Vinginevyo, msomee** sentensi **nyingine ya mfano; kwa mfano:** “*Napenda michezo*!” na ufuate hatua ulizotumia hapo juu. |
| **Soma**: Sasa nitasoma orodha ya sentensi zinazohusu hisia za jumla kuhusu wewe mwenyewe. Tafadhali nionyeshe jinsi sentensi hizi zinavyoelezea hisia zako kwa kuinua kadi ya kijani au kadi nyekundu kama ulivyofanya punde kwenye jaribio.  **Mkumbushe mshiriki kwamba kadi ya KIJANI inamaanisha “*Nakubaliana*” na NYEKUNDU inamaanisha “*Sikubaliani*”. Baada ya mshiriki kuonyesha msimamo wa kukubaliana au kutokubaliana na** sentensi **andika alama maalum kwenye nafasi inayohusika kulingana na rangi ya kadi aliyochagua.**  **[Iwapo amechagua kadi ya KIJANI]:** Sasa nataka kufahamu: unakubaliana tu kwamba **[SOMA SENTENSI]** au unakubaliana kabisa?  **[Iwapo amechagua kadi NYEKUNDU]:** Sasa nataka kufahamu: hukubaliani tu kwamba **[SOMA SENTENSI]** au hukubaliani hata kidogo? |

| **NA** | **MASWALI NA VIGEZO VYA KUCHUJIA MAJIBU** | **ALAMA MAALUMU ZA MATAMKO** | | |
| --- | --- | --- | --- | --- |
| **E01** | Kwa ujumla, naridhika na jinsi nilivyo | NAKUBALIANA KABISA  NAKUBALIANA  SIKUBALIANI  SIKUBALIANI HATA KIDOGO | **01**  **02**  **03**  **04** |  |
| **E02** | Wakati mwingine najiona kama mtu wa hovyo kabisa | NAKUBALIANA KABISA  NAKUBALIANA  SIKUBALIANI  SIKUBALIANI HATA KIDOGO | **01**  **02**  **03**  **04** |  |
| **E03** | Najiona kama ninazo sifa nzuri kadhaa | NAKUBALIANA KABISA  NAKUBALIANA  SIKUBALIANI  SIKUBALIANI HATA KIDOGO | **01**  **02**  **03**  **04** |  |
| **E04** | Naweza kufanya mambo kwa umahiri kama vile watu wengine wengi | NAKUBALIANA KABISA  NAKUBALIANA  SIKUBALIANI  SIKUBALIANI HATA KIDOGO | **01**  **02**  **03**  **04** |  |
| **E05** | Nahisi kama sina mambo mengi ambayo naweza kujivunia | NAKUBALIANA KABISA  NAKUBALIANA  SIKUBALIANI  SIKUBALIANI HATA KIDOGO | **01**  **02**  **03**  **04** |  |
| **E06** | Kwa uhakika wakati mwingine najihisi kama sina maana yoyote (useless) | NAKUBALIANA KABISA  NAKUBALIANA  SIKUBALIANI  SIKUBALIANI HATA KIDOGO | **01**  **02**  **03**  **04** |  |
| **E07** | Najihisi kama mtu mwenye thamani sawa na wengine | NAKUBALIANA KABISA  NAKUBALIANA  SIKUBALIANI  SIKUBALIANI HATA KIDOGO | **01**  **02**  **03**  **04** |  |
| **E08** | Natamani ningekuwa najithamini/kujiheshimu zaidi | NAKUBALIANA KABISA  NAKUBALIANA  SIKUBALIANI  SIKUBALIANI HATA KIDOGO | **01**  **02**  **03**  **04** |  |
| **E09** | Nikizingatia mambo yote, najiona kama sijafanikiwa (I am a failure) | NAKUBALIANA KABISA  NAKUBALIANA  SIKUBALIANI  SIKUBALIANI HATA KIDOGO | **01**  **02**  **03**  **04** |  |
| **E10** | Nina mtazamo chanya kuhusu jinsi nilivyo (positive attitude) | NAKUBALIANA KABISA  NAKUBALIANA  SIKUBALIANI  SIKUBALIANI HATA KIDOGO | **01**  **02**  **03**  **04** |  |

| 1. **DODOSO LA HALI YA AFYA KWA UJUMLA (GHQ-12)** | | | | |
| --- | --- | --- | --- | --- |
| **NA** | **MASWALI NA VIGEZO VYA KUCHUJIA MAJIBU** | **ALAMA MAALUM ZA MAJIBU YA MASWALI** | | |
| **F01** | Katika wiki mbili zilizopita, umeweza kuweka mawazo yako katika jambo ulilokuwa unafanya? | MARA CHACHE KULIKO ILIVYO KAWAIDA  HAITOFAUTIANI NA ILIVYO KAWAIDA  IMEZIDI KIDOGO KULIKO KAWAIDA  MARA NYINGI KULIKO ILIVYO KAWAIDA | **0**  **1**  **2**  **3** |  |
| **F02** | Katika wiki mbili zilizopita, umekosa usingizi kutokana na wasiwasi? | MARA CHACHE KULIKO ILIVYO KAWAIDA  HAITOFAUTIANI NA ILIVYO KAWAIDA  IMEZIDI KIDOGO KULIKO KAWAIDA  MARA NYINGI KULIKO ILIVYO KAWAIDA | **0**  **1**  **2**  **3** |  |
| **F03** | Katika wiki mbili zilizopita, umejisikia kama umekuwa na mchango muhimu katika mambo mbalimbali? | MARA CHACHE KULIKO ILIVYO KAWAIDA  HAITOFAUTIANI NA ILIVYO KAWAIDA  IMEZIDI KIDOGO KULIKO KAWAIDA  MARA NYINGI KULIKO ILIVYO KAWAIDA | **0**  **1**  **2**  **3** |  |
| **F04** | Katika wiki mbili zilizopita, umejisikia kama una uwezo wa kufanya maamuzi kuhusu mambo mbalimbali? | MARA CHACHE KULIKO ILIVYO KAWAIDA  HAITOFAUTIANI NA ILIVYO KAWAIDA  IMEZIDI KIDOGO KULIKO KAWAIDA  MARA NYINGI KULIKO ILIVYO KAWAIDA | **0**  **1**  **2**  **3** |  |
| **F05** | Katika wiki mbili zilizopita, umejisikia kama unafanya mambo kwa shinikizo wakati wote? | MARA CHACHE KULIKO ILIVYO KAWAIDA  HAITOFAUTIANI NA ILIVYO KAWAIDA  IMEZIDI KIDOGO KULIKO KAWAIDA  MARA NYINGI KULIKO ILIVYO KAWAIDA | **0**  **1**  **2**  **3** |  |
| **F06** | Katika wiki mbili zilizopita, umesikia kwamba huwezi kutatua matatizo yanayokukabili? | MARA CHACHE KULIKO ILIVYO KAWAIDA  HAITOFAUTIANI NA ILIVYO KAWAIDA  IMEZIDI KIDOGO KULIKO KAWAIDA  MARA NYINGI KULIKO ILIVYO KAWAIDA | **0**  **1**  **2**  **3** |  |
| **F07** | Katika wiki mbili zilizopita, umeweza kufurahia shughuli za kawaida za kila siku? | MARA CHACHE KULIKO ILIVYO KAWAIDA  HAITOFAUTIANI NA ILIVYO KAWAIDA  IMEZIDI KIDOGO KULIKO KAWAIDA  MARA NYINGI KULIKO ILIVYO KAWAIDA | **0**  **1**  **2**  **3** |  |
| **F08** | Katika wiki mbili zilizopita, umeweza kuyakabili matatizo yako? | MARA CHACHE KULIKO ILIVYO KAWAIDA  HAITOFAUTIANI NA ILIVYO KAWAIDA  IMEZIDI KIDOGO KULIKO KAWAIDA  MARA NYINGI KULIKO ILIVYO KAWAIDA | **0**  **1**  **2**  **3** |  |
| **F09** | Katika wiki mbili zilizopita, umejisikia mwenye kukosa raha na kupata msongo wa mawazo? | MARA CHACHE KULIKO ILIVYO KAWAIDA  HAITOFAUTIANI NA ILIVYO KAWAIDA  IMEZIDI KIDOGO KULIKO KAWAIDA  MARA NYINGI KULIKO ILIVYO KAWAIDA | **0**  **1**  **2**  **3** |  |
| **F10** | Katika wiki mbili zilizopita, umesikia hali ya kutojiamini? | MARA CHACHE KULIKO ILIVYO KAWAIDA  HAITOFAUTIANI NA ILIVYO KAWAIDA  IMEZIDI KIDOGO KULIKO KAWAIDA  MARA NYINGI KULIKO ILIVYO KAWAIDA | **0**  **1**  **2**  **3** |  |
| **F11** | Katika wiki mbili zilizopita, umejifikiria kama mtu asiye na thamani yoyote? | MARA CHACHE KULIKO ILIVYO KAWAIDA  HAITOFAUTIANI NA ILIVYO KAWAIDA  IMEZIDI KIDOGO KULIKO KAWAIDA  MARA NYINGI KULIKO ILIVYO KAWAIDA | **0**  **1**  **2**  **3** |  |
| **F12** | Katika wiki mbili zilizopita, umejisikia mwenye furaha, licha ya mambo yote yanayoendelea? | MARA CHACHE KULIKO ILIVYO KAWAIDA  HAITOFAUTIANI NA ILIVYO KAWAIDA  IMEZIDI KIDOGO KULIKO KAWAIDA  MARA NYINGI KULIKO ILIVYO KAWAIDA | **0**  **1**  **2**  **3** |  |

| **G. AINA NYINGINE ZA MATUNZO NA MSAADA** | | | | |
| --- | --- | --- | --- | --- |
| **NA** | **MASWALI NA VIGEZO VYA KUCHUJIA MAJIBU** | **ALAMA MAALUM ZA MAJIBU YA MASWALI** | | |
| **G01** | Je, umewahi kuhudhuria kikundi cha watoto au vijana wanaoishi na VVU kwa ajili ya kubadilishana uzoefu? | ndiyo  hapana  sifahamu | **1**  **2**  **8** |  |
| **G02** | Kikundi hicho (kilikuwa) kinaitwaje? | \|  \| **ndy** \| **hpn** \| **hsk** \| **SF** \| \| --- \| --- \| --- \| --- \| --- \| \| Klabu ya Ariel \| **1** \| **2** \| **6** \| **8** \| \| nyingine \| **1** \| **2** \| **6** \| **8** \|   Eleza_________________________________ | | |
| **G03** | Mara mwisho ulihudhuria wapi kikundi kama hicho? | Kituo cha Tiba ya VVU na Matunzo (CTC)  Eneo katika Jamii  nyingine ____________________________ | **1**  **2**  **3** |  |
| **G04** | Ulikuwa na umri gani ulipohudhuria kikundi kama hicho kwa mara ya mwisho? | \| umri kwa miaka \|  \|  \| \| --- \| --- \| --- \| | |  |
| **G05** | Umehudhuria kikundi kama hicho mara nyingi kiasi gani katika mwaka uliopita? | mara chache, mara moja au mbili tu  mara nyingi kiasi, kama nusu ya mikutano  mara nyingi, karibu mara zote ilipofanyika  sifahamu | **1**  **2**  **3**  **8** |  |
| **G06** | Je, kikundi hicho kina kikomo cha umri, ili wanakikundi wanapofikia umri huo waweze kuondoka kweye kikundi (na kuacha kuhudhuria)? | ndiyo  hapana  sifahamu | **1**  **2**  **8** |  |
| **G07** | Je, kuna mtu yeyote ambae unaweza kumwendea kwa ajili ya ushauri au faraja unapojisikia kukata tamaa au unapohisi kutingwa na mawazo? | ndiyo  hapana  sifahamu | **1**  **2**  **8** |  |

| **G08** | Yupo mtu ninaeweza kuzungumza nae nikijisikia kukosa raha, mwenye wasiwasi au nikiwa na msongo wa mawazo. | **Sikubaliani hata kidogo** | **Sikubaliani** | **Nakubaliana** | **Nakubaliana kabisa** | **Haihusiki** |
| --- | --- | --- | --- | --- | --- | --- |
| **G09** | Yupo mtu ninaeweza kuwasiliana nae nikihitaji kuzungumza kuhusu jambo muhimu la binafsi . | **Sikubaliani hata kidogo** | **Sikubaliani** | **Nakubaliana** | **Nakubaliana kabisa** | **Haihusiki** |
| **G010** | Majirani zangu wananiamini. | **Sikubaliani hata kidogo** | **Sikubaliani** | **Nakubaliana** | **Nakubaliana kabisa** | **Haihusiki** |
| **G011** | Ninawaamini majirani zangu. | **Sikubaliani hata kidogo** | **Sikubaliani** | **Nakubaliana** | **Nakubaliana kabisa** | **Haihusiki** |
| **G012** | Yupo mtu ninaeweza kumwendea nikihitaji kukopa hela, kupata msaada kwenda kwa daktari au kupata msaada wowote wa haraka. | **Sikubaliani hata kidogo** | **Sikubaliani** | **Nakubaliana** | **Nakubaliana kabisa** | **Haihusiki** |
| **G013** | Watu waliopo katika maisha yangu wananipa taarifa, mapendekezo au mwongozo pale ninapohitaji. | **Sikubaliani hata kidogo** | **Sikubaliani** | **Nakubaliana** | **Nakubaliana kabisa** | **Haihusiki** |
| **G014** | Majirani zangu wanajisikia huru kuzungumza na mimi kuhusu matatizo yao. | **Sikubaliani hata kidogo** | **Sikubaliani** | **Nakubaliana** | **Nakubaliana kabisa** | **Haihusiki** |

| Muda wa kumaliza mahojiano: |  |  |  | : |  |  |  |
| --- | --- | --- | --- | --- | --- | --- | --- |
|  | | saa | | dakika | | | |

| **H.Hitimisho** | | | | |
| --- | --- | --- | --- | --- |
| **NO** | **QUESTIONS AND FILTERS** | **ALAMA MAALUM ZA MASWALI** | | **KURUKA SWALI** |
| **H01** | Je, mshiriki alikatisha mahojiano? | NDIO  HAPANA | **1**  **2** | **>> Q2** |
| **H01A** | Kwanini mshiriki alikatisha mahojiano mapema? | AMESHIMAMISHA KWA MUDA TU. ANGEPENDELEA KUENDELEA MUDA MWINGINE.  AMECHOKA  ANASHUGHULI NYINGI SANA/HANA MUDA  ALIKWAZIKA NA MASWALI  ALIPATA WASIWASI WA MDAHILI AU LENGO LA UTAFITI  OTHER HAJISIKII KUENDELEA NA UTAFITI ­­­­­MENGINEYO ______ _ __________  SIJUI | **1**  **2**  **3**  **4**  **5**  **6**  **7**  **8** |  |
| **H02** | Je, kulikuwa na wadihili wengine wakati wa mahojiano? | NDIO  HAPANA | **1**  **2** | **>> Q3** |
| **H02A** | Tafadhali andika namba zote za utambulisho za wafanyakazi wote waliokuwepo Zaidi ya mtu anayedahili | **NAMBA YA UTAMBULISHO___________________________________**  **_____________________________________________** | |  |
| **H03** | Je, palikuwa na mtu mwingine yoyote kwenye udahili? | NDIO  HAPANA | **1**  **2** | **>> Q4** |
| **H03A** | Upi ni uhusiano wao na mshiriki? | **Uhusiano__________________________________**  **______________________________________________** | |  |
| **H04** | Una uhakika sana, una uhakika kiasi ama hauna uhakika sana na ubora wa ujumla na ukweli wa majibu ya mshiriki huyu? | NINA UHAKIKA SANA  NINA UHAKIKA KIASI  SINA UHAKIKA | **1**  **2**  **3** | **>> Mwisho** |
| **H04A** | Nini kinapelekea usiwe na uhakika? | **Fafanua___________________________________**  ___________________________________________________ |  |  |
