# Supplementary material for: Adolescent support club attendance and self-efficacy associated with HIV treatment outcomes in Tanzania
Source: PLOS Glob Public Health. 2022 Oct 3;2(10):e0000065. doi: 10.1371/journal.pgph.0000065 (PMC10021176; doi:10.1371/journal.pgph.0000065)
Supplement: S1 Table — (DOCX) [file pgph.0000065.s003.docx]

**Outcomes among adolescents missing viral load (n=645)**

|  | **All adolescents (n=645)** | **Has viral load (n=536)** | **Missing viral load**  **(n=109)** | **p-value*** | |
| --- | --- | --- | --- | --- | --- |
|  | **N (%) or**  **Median (inter-quartile range [IQR])** | | |  |  |
| **Good visit adherence**  (not missed >=1 ART refill visit by 5+ days) | 72 (11.2) | 68 (12.7) | 4 (3.7) | 0.006 | |
| **Retention** | 454 (70.2) | 417 (78.0) | 35 (32.1) | <0.0001 | |

Adolescents missing viral load measures are 2-3 times more likely to have poor adherence and not be retained. Sensitivity analysis below shows regression results under different assumptions, where all missing values are imputed as “suppressed”, “not suppressed”, or 2:1 not suppressed:suppressed (the scenario most consistent with table above), where those assigned to not vs suppressed status are randomly selected from all n=109 missing.

**Sensitivity Analysis: Facility-adjusted* logistic regression modelling of associations between adolescent club attendance and viral suppression among all adolescents attending study sites and the subset of adolescents enrolled and interviewed**

|  | **Odds ratio [OR]**  **(95% CI)** | **p-value** | **OR (95% CI)** | **p-value** | **OR (95% CI)** | **p-value** | **OR (95% CI)** | **p-value** |
| --- | --- | --- | --- | --- | --- | --- | --- | --- |
| **All adolescent model** | **n=536 non-missing** | | **n=645, n=109 missing set to “1” virally suppressed** | | **n=645, n=109 missing set to “0” NOT virally suppressed** | | **n=645, n=109 missing set to “0” (n=73) or “1” (n=36), randomly at ratio 2:1** | |
| **Club attendance**  None  1-9 club meetings  >10 club meetings | 1.00  0.70 (0.41, 1.20)  1.12 (0.66, 1.88) | 0.19  0.68 | 1.00  0.40 (0.24, 0.65)  0.53 (0.32, 0.86) | <0.0001  0.011 | 1.00  1.72 (1.07, 2.79)  3.47 (2.16, 5.58) | 0.026  <0.0001 | 1.00  1.23 (0.78, 1.93)  2.07 (1.32, 3.23) | 0.37  0.001 |
| Male sex (ref: F) | 0.97 (0.68, 1.38) | 0.87 | 0.83 (0.59, 1.15) | 0.27 | 0.80 (0.57, 1.12) | 0.20 | 0.86 (0.62, 1.19) | 0.35 |
| Age in years | 0.93 (0.87, 1.00) | 0.045 | 1.01 (0.95, 1.08) | 0.045 | 0.90 (0.85, 0.96) | 0.001 | 0.93 (0.87, 0.98) | 0.013 |
| Years on ART | 0.92 (0.87, 0.97) | 0.002 | 0.92 (0.87, 0.97) | 0.002 | 0.94 (0.89, 0.99) | 0.014 | 0.94 (0.90, 0.99) | 0.027 |
| **Subset interviewed adolescents** | **n=148 non-missing** | | **n=154, n=6 missing set to “1” virally suppressed** | | **n=154, n=6 missing set to “0” NOT virally suppressed** | | **NA** | |
| **Club attendance** (Nov2016-Oct2018)  None  1-9 club meetings  >10 club meetings | 1.00  0.54 (0.11, 2.73)  2.42 (0.49, 11.81) | 0.46  0.28 | 1.00  0.29 (0.07, 1.16)  1.06 (0.26, 4.29) | 0.08  0.93 | 1.00  1.42 (0.33, 6.15)  6.22 (1.45, 26.67) | 0.64  0.014 | — | — |
| **Self-efficacy tertile**  Low (ref)  Medium  High | 1.00  2.12 (0.78, 5.73)  3.04 (1.08, 8.60) | 0.14  0.036 | 1.00  2.07 (0.78, 5.53)  2.71 (1.08, 7.31) | 0.14  0.048 | 1.00  2.07 (0.78, 5.50)  2.51 (0.93, 6.82) | 0.14  0.07 | — | — |
| Male sex (ref: F) | 0.67 (0.30, 1.54) | 0.35 | 0.66 (0.29, 1.46) | 0.30 | 0.68 (0.31, 1.52) | 0.35 | — | — |
| Age in years | 1.01 (0.86, 1.19) | 0.89 | 1.01 (0.86, 1.19) | 0.91 | 1.01 (0.86, 1.18) | 0.91 | — | — |
| Years on ART | 0.89 (0.78, 1.01) | 0.069 | 0.88 (0.78, 1.00) | 0.06 | 0.91 (0.81, 1.03) | 0.15 | — | — |
| **Poor adherence indicator**  (missed ARV dose in past 7 days) | 0.23 (0.09, 0.57) | 0.001 | 0.24 (0.10, 0.57) | 0.001 | 0.25 (0.10, 0.60) | 0.002 | — | — |
| **Guardian/treatment supporter attends clinic with adolescent**  (ref: adolescent attends on own) | 3.29 (1.17, 9.22) | 0.024 | 2.92 (1.07, 7.93) | 0.036 | 3.39 (1.24, 9.27) | 0.017 | — | — |
| * All facilities included in model as dummy variable. | | | | | | | | |
